# Supplementary material for: Personal, social, and natural co-exposure pattern and plasma proteins in cardiometabolic diseases
Source: Nat Commun. 2025 Nov 25;16:10498. doi: 10.1038/s41467-025-65516-2 (PMC12647215; doi:10.1038/s41467-025-65516-2)
Supplement: Supplementary file 1 — Supplementary Information [file 41467_2025_65516_MOESM1_ESM.pdf]

SUPPLEMENTARY INFORMATION

Personal, Social, and Natural Environmental Exposures and Plasma Proteins in Cardiometabolic Diseases

Supplementary Methods 2
Supplementary Figures 4
Supplementary Tables 11
Supplementary Reference 60

Supplementary Methods

UK Biobank recruitment

UK Biobank (UKB) is a prospective cohort study that recruited 500,000 participants across the UK, with assessments conducted at 22 centers in Scotland, England, and Wales. Recruitment was centrally coordinated through population-based registers, such as those held by the NHS, identifying and inviting eligible individuals living within a reasonable distance of an assessment center. Each center aimed to recruit as many local participants as possible over six months to a year, depending on population density and transport accessibility, before relocating to maximize nationwide coverage. Upon consenting to participate, individuals attended a nearby assessment center for baseline data collection, physical measurements, and biological sampling. Initially, UKB planned for 35 assessment centers across the UK, and 22 centers were ultimately established during baseline assessments from 2006 to 2010. The assessment process lasted 2–3 hours and comprised five key components: written consent, touchscreen questionnaires, a face-to-face interview with a study nurse, physical measurements, and biological sample collection (blood, urine, and saliva)[1]. The sample size for each assessment center in this study is shown below.

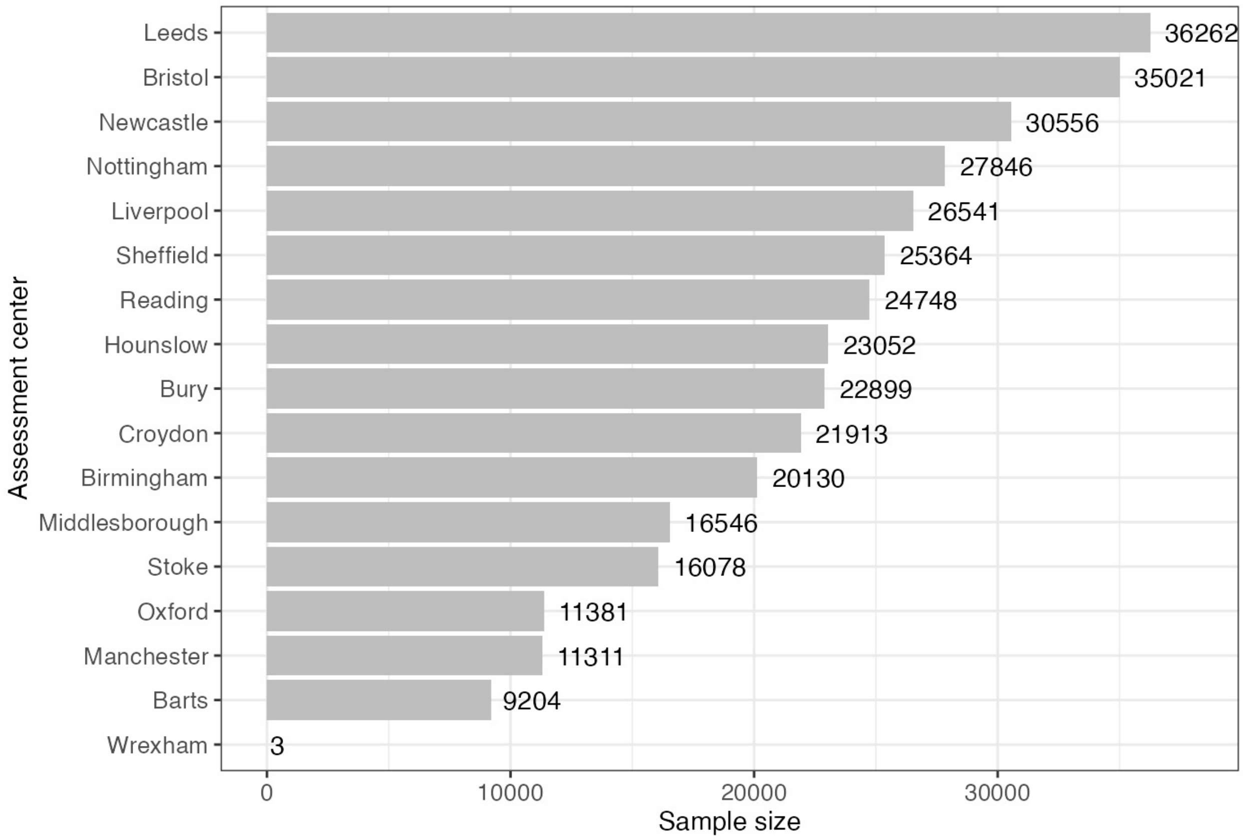

Figure 1 for Supplementary Methods

Air pollutant exposures

Ambient air pollutant estimates were collected by the UK Biobank during 2005–2007 and 2010. Annual concentration data for PM2.5, PM2.5–10, and NOx were available only for 2010, while NO2 data spanned 2005–2007 and 2010, and PM10 data covered 2007 and 2010. To ensure consistency, we selected 2010 concentrations for all pollutants as the exposure metric. The 2010 annual mean air pollution concentrations were derived using a land use regression (LUR) model, which integrated participants' residential addresses from the baseline visit and monitoring data from the European Study of Cohorts for Air Pollution Effects (ESCAPE) for the period January 26, 2010, to January 18, 2011[1]. Figure2 for Supplementary Methods presents box plots depicting the distributions of environmental exposure factors.

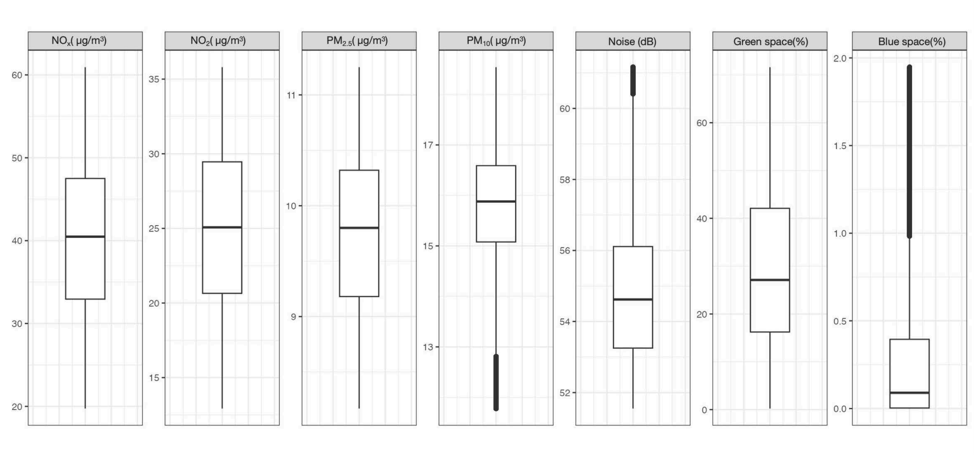

Ascertainment of outcomes

Heart disease, cerebrovascular disease, renal disease, and type 2 diabetes were classified based on the 10th Edition of the International Classification of Diseases (ICD-10) (see table below). Detailed information, including admission dates and causes, was obtained from UK Biobank through hospital records, NHS Information Centre (England and Wales) death certificates, primary care data, and the NHS Central Register (Scotland). Follow-up data on cardiometabolic disease events and mortality were tracked via electronic linkage to hospital admissions and death registry records across England, Wales, and Scotland.

Table for Supplementary Methods

**Outcome**	**ICD-10 Codes**
Heart disease	I20-I25, I48, I50
Cerebrovascular disease	I60-I69
Renal disease	N17, N18, I12
Diabetes	E11

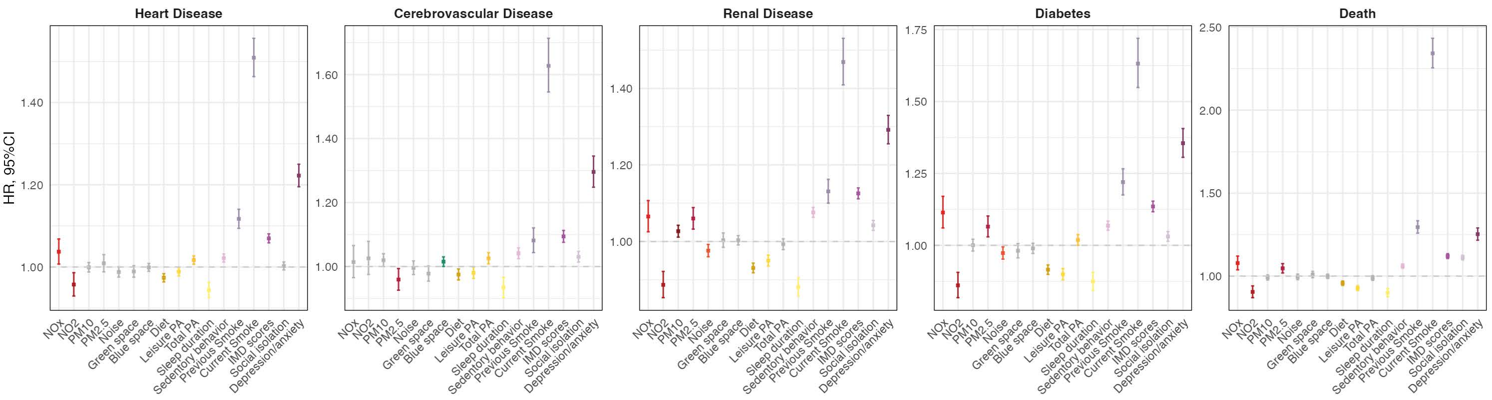

Supplementary Fig. 1 Associations between exposures and cardiometabolic diseases (CMDs) across the overall population, analyzed using multi-exposure multivariable Cox proportional hazards regression models, adjusted for age, sex, ethnicity, alcohol consumption status, and BMI. Each exposure was standardized prior to modeling. Circular markers represent HR point estimates for each association between an individual exposure and an outcome. Vertical error bars represent 95% confidence intervals (CIs). The horizontal dashed line denotes the null value of HR = 1. Colors denote the type of exposure, with gray denoting exposures with an adjusted p-value > 0.05. Statistical significance was assessed using two-sided Wald tests. Source data are provided. The sample sizes (independent UK Biobank participants) for the cardiometabolic outcomes were n = 336,778 for heart disease, n = 353,264 for cerebrovascular disease, n = 353,892 for renal disease, n = 341,529 for diabetes, and n = 358,872 for death. BMI, body mass index; PM, particulate matter; IMD, Index of Multiple Deprivation; PA, physical activity.

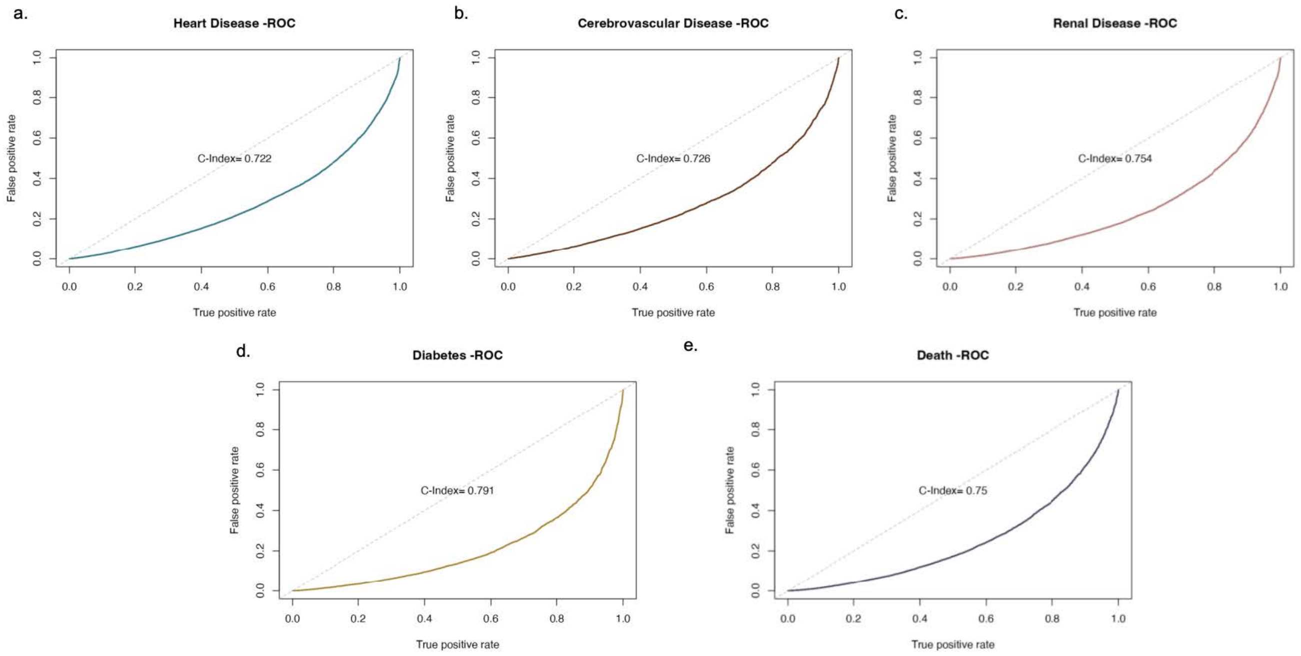

Supplementary Fig. 2 ROC Curves and C-Indices for Different Cardiometabolic Diseases Using XGBoost. XGBoost survival models with Cox proportional hazards objective were trained on 70% of the study population and evaluated on the remaining 30%. The diagonal dashed line represents random classification. The sample sizes (independent UK Biobank participants) for the cardiometabolic outcomes were n = 336,778 for heart disease, n = 353,264 for cerebrovascular disease, n = 353,892 for renal disease, n = 341,529 for diabetes, and n = 358,872 for death.

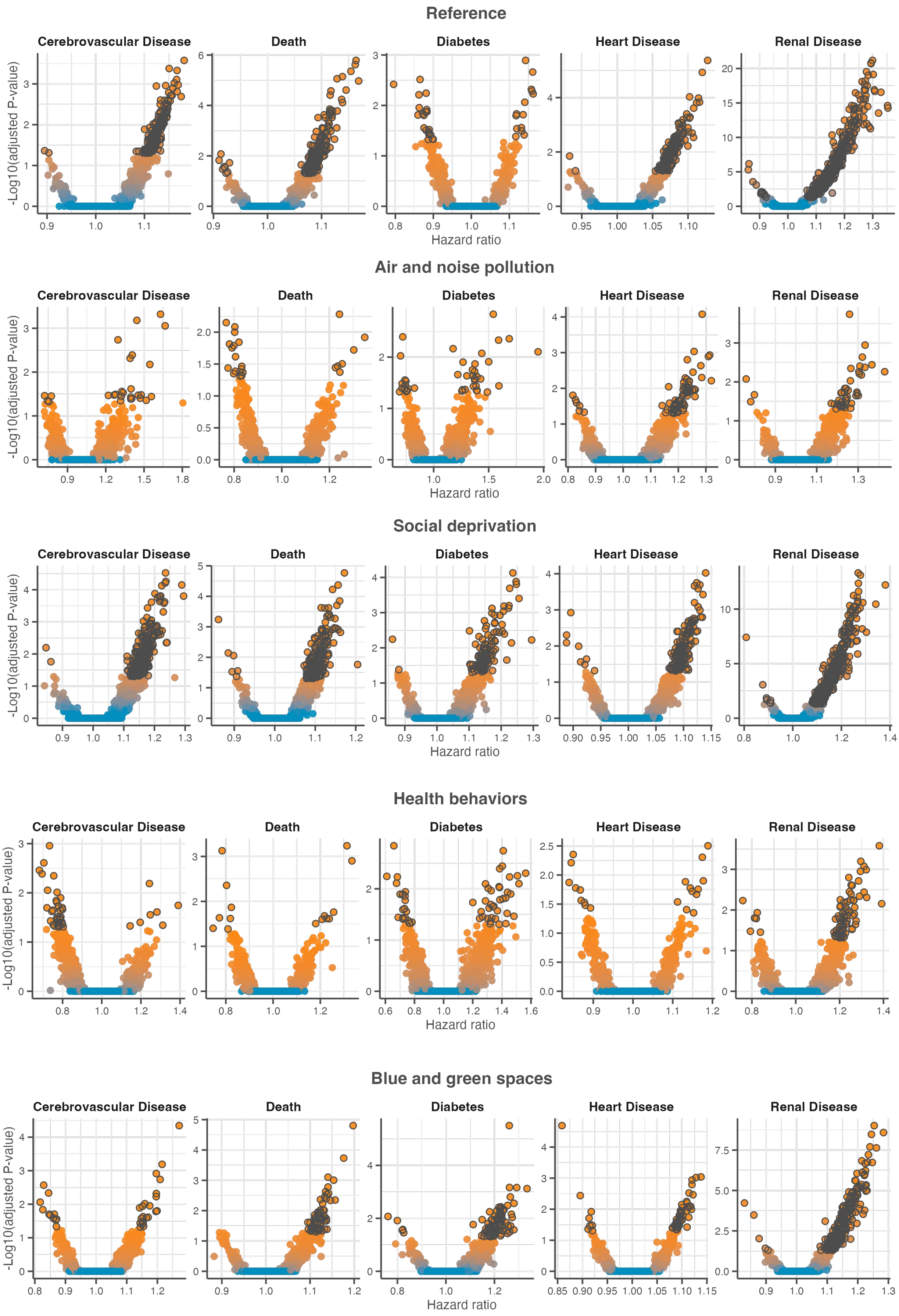

Supplementary Fig. 3 Association between proteins and cardiometabolic diseases across different exposure pattern subgroups estimated using Cox proportional hazards models. Statistical significance was assessed using two-sided Wald tests with Bonferroni correction for multiple comparisons (exact P values are provided in Supplementary Data 2). The sample sizes (independent UK Biobank participants) for the cardiometabolic outcomes were n = 35,056 for heart disease, n = 37,025 for cerebrovascular disease, n = 36,963 for renal disease, n = 35,754 for diabetes, and n = 37,687 for death. The color of each point represents the adjusted P value, and points with adjusted P < 0.05 are highlighted with an outline.

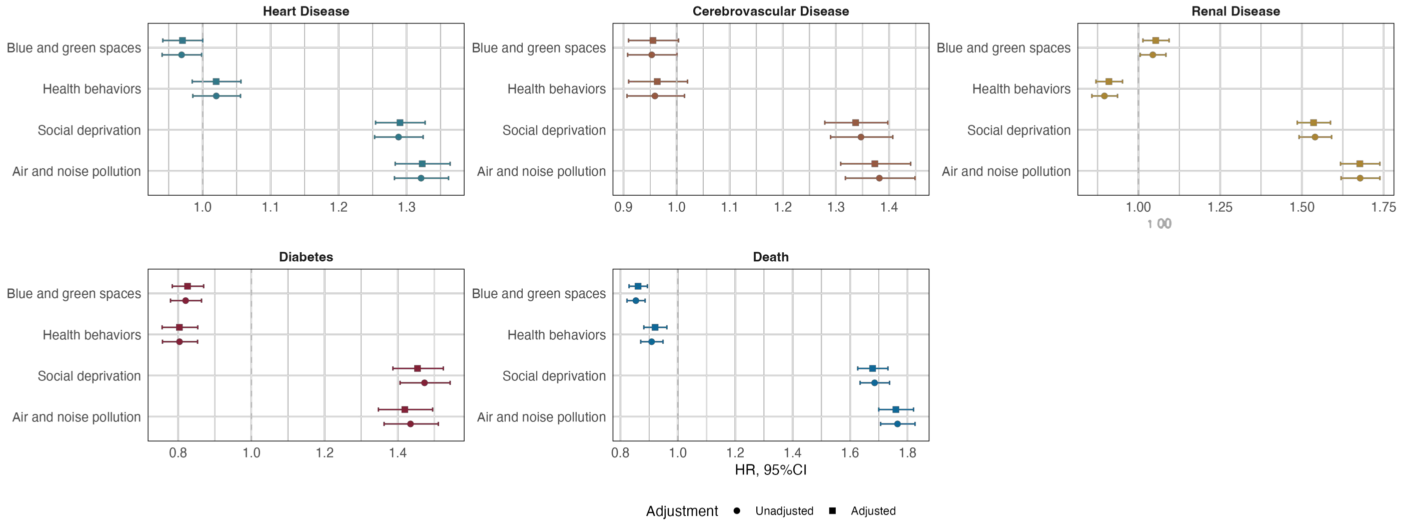

Supplementary Fig. 4 Association between proteomics signatures and cardiometabolic diseases with and without adjusted for co-exposure pattern using Cox proportional hazards models. Circular markers represent hazard ratio (HR) point estimates from models adjusted for age, sex, ethnicity, alcohol consumption status, and BMI. Square markers represent HR point estimates from models further adjusted for the corresponding co-exposure pattern in addition to the aforementioned covariates. Error bars represent 95% confidence intervals (CIs). The dashed line denotes the null value (HR = 1). The sample sizes (independent UK Biobank participants) for the cardiometabolic outcomes were n = 35,056 for heart disease, n = 37,025 for cerebrovascular disease, n = 36,963 for renal disease, n = 35,754 for diabetes, and n = 37,687 for death.

Disease	Air and noise pollution	Social deprivation	Healthy Behavior	Blue and Green Space
**Heart disease**		GDF15<br>CXCL17<br>PIGR<br>IGFBP4<br>ACVRL1	TNFRSF12A<br>FABP3<br>HSPB6<br>RBP2<br>EDA2R	
**Cerebrovascular disease**	CDCP1<br>TFF2<br>IGFBP4<br>GDF15	PIGR<br>GDF15<br>CXCL17<br>ALPP<br>IGFBP4		
**Renal disease**	IGFBP4<br>GDF15<br>TNFRSF12A<br>TFF2<br>REN	IGFBP4<br>GDF15<br>ACVRL1<br>PIGR<br>TFF1	CD38<br>PTGDS<br>ADM<br>GFRA1<br>CLMP	
**Diabetes**	CDCP1<br>FGF21<br>SIGLEC7<br>GDF15<br>CXCL17	PIGR<br>FGF21<br>GDF15<br>CXCL17<br>CDCP1	PDZK1<br>CA6<br>CA14<br>SDC1<br>ACP5	
**Death**	CDCP1<br>TFF2<br>CEACAM5<br>CXCL17<br>IGFBP4	GDF15<br>PIGR<br>CXCL17<br>IGFBP4<br>CEACAM5	ITGA11<br>ITGAV<br>ADM<br>MMP7<br>GFRA1	MMP12<br>HPGDS<br>ITGAV<br>ADGRG2

Air and noise pollution | Social deprivation | Healthy Behavior | Blue and Green Space

Supplementary Fig. 5 Exposure Patterns Corresponding to the Top Five Proteins with the Highest Mediated Proportions for Each Disease. Mediation proportions were estimated using a regression-based mediation approach with a Cox outcome model and a linear mediator model. P-values for the natural indirect effects were calculated using two-sided Z-tests based on the delta method and adjusted for multiple comparisons using the Bonferroni method. After correction and exclusion of inconsistent mediation, only the top five proteins with the highest mediated proportions and statistically significant adjusted p-values ($p < 0.05$) for each exposure pattern–outcome pair are shown. The background colour of each cell represents the exposure pattern. Protein names highlighted in red within cells indicate common proteins that exhibited both high mediation proportions and high frequency of occurrence for the same disease. The sample sizes (independent UK Biobank participants) for the cardiometabolic outcomes were n =35,056 for heart disease, n = 37,025 for cerebrovascular disease, n = 36,963 for renal disease, n = 35,754 for diabetes, and n = 37,687 for death. Full protein names are listed in Supplementary Data 1.

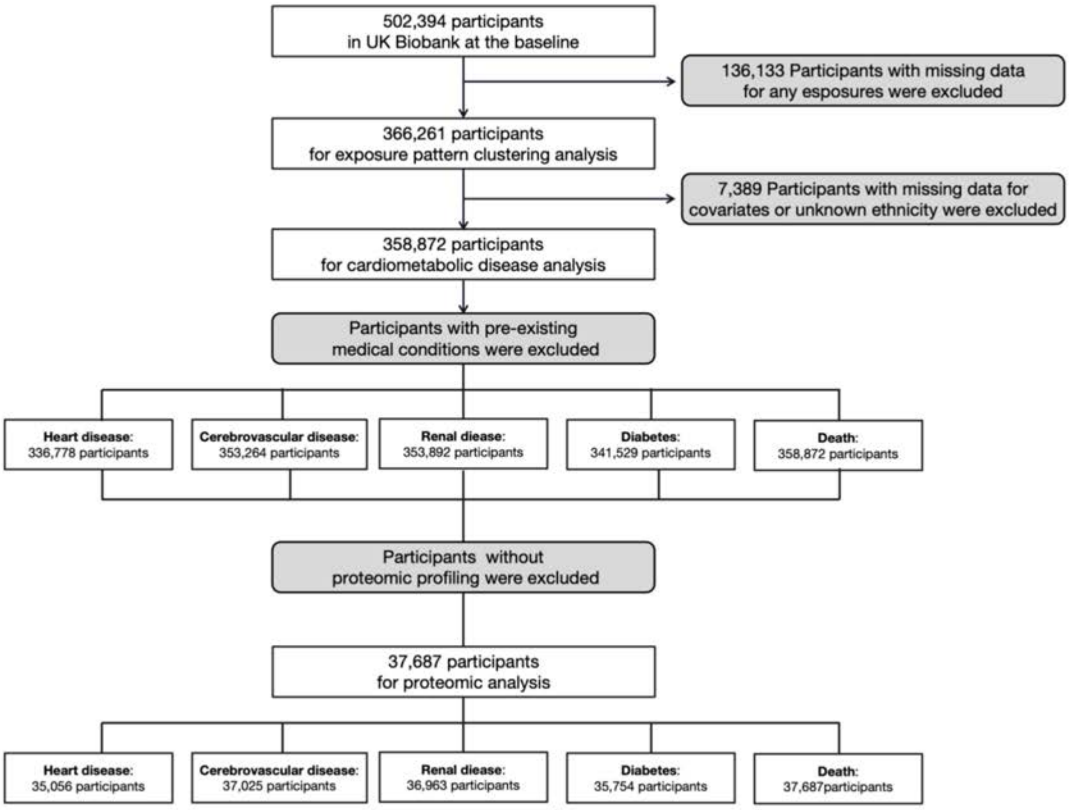

Supplementary Fig. 6 Participant Inclusion and Exclusion Flowchart from the UK Biobank.

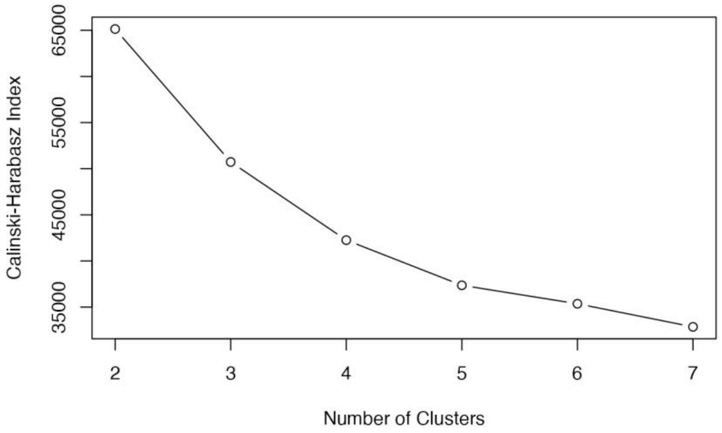

Supplementary Fig. 7 CH-index with various numbers of clusters in K-prototypes analysis

Supplementary Table 1 Basic Characteristics of Participants Included in the Analyses

Analysis data set	Clustering	Heart disease	Cerebrovascular disease	Renal Diease	Diabetes	Death	Proteomic profiling
number of participants	366261	336778	353264	353892	341529	358872	37687
Age [mean (SD)]	56.43 (8.07)	56.41 (8.06)	56.06 (8.06)	56.35 (8.07)	56.24 (8.08)	56.34 (8.06)	56.65 (8.17)
Male (%)	169867 (46.4)	166086 (46.3)	150736 (44.8)	162826 (46.1)	155141 (45.4)	163531 (46.2)	17543 (46.5)
Ethnicity (%)							
White	347657 (94.9)	341723 (95.2)	320655 (95.2)	336354 (95.2)	326334 (95.6)	337025 (95.2)	35594 (94.4)
Nonwhite	17573 (4.8)	16123 (4.8)	16910 (4.8)	16867 (4.8)	15195 (4.4)	17149 (4.8)	2093 (5.6)
Unknown	1031 (0.3)	/	/	/	/	/	/
BMI, kg/m2 [mean (SD)]	27.34 (4.72)	27.34 (4.72)	27.22 (4.67)	27.32 (4.71)	27.14 (4.57)	27.31 (4.70)	27.36 (4.71)
Alcoholic consumption (%)							
Never	14208 (3.9)	13773 (3.8)	12679 (3.8)	13468 (3.8)	12487 (3.7)	13442 (3.8)	1479 (3.9)
Previous	12219 (3.3)	11810 (3.3)	10557 (3.1)	11398 (3.2)	10658 (3.1)	11521 (3.3)	1372 (3.6)
Current	339834 (92.8)	333289 (92.9)	313542 (93.1)	328398 (93.0)	318384 (93.2)	328929 (92.9)	34836 (92.4)
NO2, μg/m3 [mean (SD)]	26.48 (7.56)	26.46 (7.56)	26.46 (7.55)	26.47 (7.56)	26.41 (7.55)	26.47 (7.56)	26.66 (7.67)
NOx, μg/m3 [mean (SD)]	43.65 (15.38)	43.58 (15.34)	43.59 (15.34)	43.62 (15.36)	43.52 (15.31)	43.62 (15.36)	43.97 (15.60)
PM2.5, μg/m3 [mean (SD)]	9.96 (1.04)	9.96 (1.04)	9.96 (1.04)	9.96 (1.04)	9.95 (1.04)	9.96 (1.04)	9.98 (1.05)
PM10, μg/m3 [mean (SD)]	16.19 (1.88)	16.19 (1.88)	16.19 (1.88)	16.19 (1.88)	16.18 (1.88)	16.19 (1.88)	16.21 (1.89)
24- hour noise, dB [mean (SD)]	56.02 (4.26)	56.02 (4.25)	56.02 (4.25)	56.02 (4.25)	56.01 (4.24)	56.02 (4.25)	56.05 (4.30)
Greenspace percentage [mean (SD)]	35.64 (23.38)	35.65 (23.44)	35.68 (23.41)	35.66 (23.41)	35.78 (23.47)	35.67 (23.40)	35.63 (23.40)
Bluespace percentage [mean (SD)]	0.88 (2.91)	0.88 (2.90)	0.88 (2.92)	0.89 (2.92)	0.89 (2.93)	0.88 (2.91)	0.84 (2.71)
Diet Score [mean (SD)]	2.45 (1.16)	2.45 (1.16)	2.45 (1.16)	2.45 (1.16)	2.45 (1.16)	2.45 (1.16)	2.45 (1.15)
Leisure Physical Activity, MET [mean (SD)]	1180.69 (1478.89)	1189.69 (1481.03)	1186.95 (1479.95)	1187.03 (1480.71)	1196.87 (1483.15)	1184.63 (1480.43)	1175.07 (1477.46)
Total Physical Activity, MET [mean (SD)]	2568.99 (3025.75)	2582.67 (3027.68)	2577.98 (3028.66)	2579.10 (3030.04)	2594.20 (3035.30)	2574.38 (3028.09)	2559.78 (2982.28)
Sedentory behavior, hours [mean (SD)]	3.86 (2.09)	3.82 (2.06)	3.85 (2.08)	3.85 (2.08)	3.82 (2.05)	3.86 (2.08)	3.88 (2.10)
Index of Multiple Deprivation [mean (SD)]	17.07 (13.57)	16.84 (13.39)	16.96 (13.48)	16.98 (13.51)	16.81 (13.38)	17.01 (13.53)	17.55 (13.84)
Social isolation index [mean (SD)]	0.70 (0.74)	0.69 (0.73)	0.69 (0.73)	0.69 (0.73)	0.69 (0.73)	0.70 (0.74)	0.70 (0.73)
Anxiety or Depression (%)	89456 (24.4)	81056 (24.1)	85417 (24.2)	85884 (24.3)	82076 (24.0)	87289 (24.3)	9346 (24.8)
Appropriate Sleep (%)	144252 (39.4)	134545 (40.0)	139981 (39.6)	140181 (39.6)	136088 (39.8)	141704 (39.5)	14745 (39.1)
Smoke(%)							
Never	200766 (54.8)	188007 (55.8)	194679 (55.1)	194539 (55.0)	189189 (55.4)	196968 (54.9)	20459 (54.3)
Previous	129102 (35.2)	115494 (34.3)	123885 (35.1)	124239 (35.1)	118612 (34.7)	126387 (35.2)	13467 (35.7)
Current	36393 (9.9)	33277 (9.9)	34700 (9.8)	35114 (9.9)	33728 (9.9)	35517 (9.9)	3761 (10.0)
Outcome incidence (%)	/	43173 (12.8)	14863 (4.2)	24720 (7.0)	13940 (4.1)	23902 (6.7)	/

Supplementary Table 2 Basic Characteristics of Participants by Different Exposure Patterns

	Reference	Air and Noise Pollution	Social Deprivation	Blue and Green space	Health Behaviors
NO2, μg/m3 [mean (SD)]	26.14 (3.85)	39.95 (9.32)	33.23 (4.70)	18.09 (3.36)	25.62 (5.26)
NOx, μg/m3 [mean (SD)]	42.01 (6.45)	79.05 (23.42)	54.90 (9.43)	28.60 (6.05)	41.52 (9.38)
PM2.5, μg/m3 [mean (SD)]	9.95 (0.51)	11.52 (1.24)	10.92 (0.81)	8.78 (0.46)	9.87 (0.74)
PM10, μg/m3 [mean (SD)]	16.41 (1.46)	18.96 (1.68)	16.88 (1.44)	14.51 (1.63)	16.21 (1.61)
24- hour noise, dB [mean (SD)]	54.84 (2.58)	68.08 (3.87)	55.84 (2.64)	55.54 (3.60)	55.35 (3.24)
Greenspace percentage [mean (SD)]	27.15 (13.81)	27.42 (18.58)	21.11 (12.27)	66.53 (18.88)	33.55 (19.76)
Bluespace percentage [mean (SD)]	0.61 (1.76)	1.02 (2.78)	0.59 (2.01)	1.63 (4.73)	0.78 (2.40)
Diet Score [mean (SD)]	2.42 (1.14)	2.48 (1.18)	2.34 (1.20)	2.47 (1.13)	2.71 (1.12)
Leisure Physical Activity, MET [mean (SD)]	844.78 (797.77)	1006.08 (1226.01)	710.87 (848.23)	1170.32 (1130.16)	3802.18 (2634.38)
Total Physical Activity, MET [mean (SD)]	1756.43 (1666.49)	2479.16 (2817.93)	2085.64 (2285.03)	2194.39 (2215.17)	8142.41 (4682.74)
Sedentory behavior, hours [mean (SD)]	3.81 (1.97)	3.88 (2.21)	4.33 (2.48)	3.71 (1.95)	3.44 (1.74)
Index of Multiple Deprivation [mean (SD)]	11.78 (7.63)	21.64 (14.54)	32.75 (15.35)	12.13 (8.82)	15.31 (11.47)
Social isolation index [mean (SD)]	0.61 (0.69)	0.79 (0.77)	0.96 (0.80)	0.65 (0.71)	0.55 (0.67)
Anxiety or Depression [n (%)]	35349 (23.5)	5648 (26.6)	23438 (31.2)	18517 (21.9)	6504 (18.8)
Appropriate Sleep [n (%)]	57632 (38.3)	8046 (37.9)	27070 (36.0)	33002 (38.9)	18502 (53.6)
Current smoke [n (%)]	12180 (8.1)	2689 (12.7)	12169 (16.2)	6079 (7.2)	3276 (9.5)

Supplementary Table 3 GO Enrichment Analysis Results for the Exposure Pattern Proteomic Signature, assessed using a one-sided Fisher's exact test with Bonferroni correction for multiple testing

Expposure pattern	ONTOLOGY	ID	Description	GeneRatio	BgRatio	RichFactor	FoldEnrichment	p.adjust	qvalue	geneID	Count
Air and noise pollution	BP	GO:1905523	positive regulation of macrophage migration	4/102	25/18888	0.1600	29.6282	0.0201	0.0005	CCL3/CX3CL1/CXCL17/RARRES2	4
Air and noise pollution	BP	GO:0035902	response to immobilization stress	4/102	26/18888	0.1538	28.4887	0.0237	0.0005	GAL/REN/SOD2/TFF1	4
Air and noise pollution	BP	GO:0048245	eosinophil chemotaxis	4/102	27/18888	0.1481	27.4336	0.0277	0.0006	CCL11/CCL16/CCL3/CX3CL1	4
Air and noise pollution	BP	GO:0072677	eosinophil migration	4/102	31/18888	0.1290	23.8937	0.0488	0.0009	CCL11/CCL16/CCL3/CX3CL1	4
Air and noise pollution	BP	GO:0002548	monocyte chemotaxis	8/102	70/18888	0.1143	21.1630	0.0000	0.0000	CCL11/CCL16/CCL3/CX3CL1/CXCL17/IL6/SLAMF8/TNFRSF11A	8
Air and noise pollution	BP	GO:1905521	regulation of macrophage migration	5/102	44/18888	0.1136	21.0428	0.0083	0.0003	CCL3/CX3CL1/CXCL17/RARRES2/SLAMF8	5
Air and noise pollution	BP	GO:1905517	macrophage migration	5/102	60/18888	0.0833	15.4314	0.0389	0.0008	CCL3/CX3CL1/CXCL17/RARRES2/SLAMF8	5
Air and noise pollution	BP	GO:0071347	cellular response to interleukin-1	9/102	110/18888	0.0818	15.1508	0.0000	0.0000	CCL11/CCL16/CCL3/CX3CL1/CXCL8/HYAL1/IL1R1/IL6/SFRP1	9
Air and noise pollution	BP	GO:0070555	response to interleukin-1	11/102	138/18888	0.0797	14.7604	0.0000	0.0000	CCL11/CCL16/CCL3/CD38/CX3CL1/CXCL8/HYAL1/IL1R1/IL6/SFRP1/TNFRSF11A	11
Air and noise pollution	BP	GO:0070098	chemokine-mediated signaling pathway	7/102	93/18888	0.0753	13.9380	0.0015	0.0001	CCL11/CCL16/CCL3/CX3CL1/CXCL13/CXCL8/TFF2	7
Air and noise pollution	BP	GO:1990868	response to chemokine	7/102	101/18888	0.0693	12.8340	0.0027	0.0001	CCL11/CCL16/CCL3/CX3CL1/CXCL13/CXCL8/TFF2	7
Air and noise pollution	BP	GO:1990869	cellular response to chemokine	7/102	101/18888	0.0693	12.8340	0.0027	0.0001	CCL11/CCL16/CCL3/CX3CL1/CXCL13/CXCL8/TFF2	7
Air and noise pollution	BP	GO:0044344	cellular response to fibroblast growth factor stimulus	8/102	116/18888	0.0690	12.7708	0.0005	0.0000	CXCL13/CXCL8/FGF21/FGF23/FGFBP1/HYAL1/SFRP1/SMOC2	8
Air and noise pollution	BP	GO:0046849	bone remodeling	6/102	90/18888	0.0667	12.3451	0.0200	0.0005	CD38/EPHA2/IL6/SFRP1/TNFRSF11A/TPP1	6
Air and noise pollution	BP	GO:0097530	granulocyte migration	10/102	154/18888	0.0649	12.0244	0.0000	0.0000	CCL11/CCL16/CCL3/CX3CL1/CXCL13/CXCL17/CXCL8/IL1R1/RARRES2/SLAMF8	10
Air and noise pollution	BP	GO:0071774	response to fibroblast growth factor	8/102	124/18888	0.0645	11.9469	0.0008	0.0000	CXCL13/CXCL8/FGF21/FGF23/FGFBP1/HYAL1/SFRP1/SMOC2	8

Supplementary Table 3 GO Enrichment Analysis Results for the Exposure Pattern Proteomic Signature, assessed using a one-sided Fisher's exact test with Bonferroni correction for multiple testing

Expposure pattern	ONTOLOGY	ID	Description	GeneRatio	BgRatio	RichFactor	FoldEnrichment	p.adjust	qvalue	geneID	Count
Air and noise pollution	BP	GO:0002690	positive regulation of leukocyte chemotaxis	6/102	93/18888	0.0645	11.9469	0.0241	0.0005	CCL3/CXCL13/CXCL17/CXCL8/IL6/RARRES2	6
Air and noise pollution	BP	GO:0071621	granulocyte chemotaxis	8/102	128/18888	0.0625	11.5735	0.0010	0.0000	CCL11/CCL16/CCL3/CX3CL1/CXCL13/CXCL17/CXCL8/RARRES2	8
Air and noise pollution	BP	GO:1990266	neutrophil migration	8/102	129/18888	0.0620	11.4838	0.0011	0.0000	CCL11/CCL16/CCL3/CX3CL1/CXCL13/CXCL8/IL1R1/SLAMF8	8
Air and noise pollution	BP	GO:0002688	regulation of leukocyte chemotaxis	7/102	123/18888	0.0569	10.5385	0.0100	0.0003	CCL3/CXCL13/CXCL17/CXCL8/IL6/RARRES2/SLAMF8	7
Air and noise pollution	BP	GO:0002687	positive regulation of leukocyte migration	8/102	149/18888	0.0537	9.9424	0.0032	0.0001	CCL3/CX3CL1/CXCL13/CXCL17/CXCL8/IL1R1/IL6/RARRES2	8
Air and noise pollution	BP	GO:0097529	myeloid leukocyte migration	12/102	239/18888	0.0502	9.2976	0.0000	0.0000	CCL11/CCL16/CCL3/CX3CL1/CXCL13/CXCL17/CXCL8/IL1R1/IL6/RARRES2/SLAMF8/TNFRSF11A	12
Air and noise pollution	BP	GO:0050921	positive regulation of chemotaxis	7/102	142/18888	0.0493	9.1284	0.0258	0.0006	CCL3/CXCL13/CXCL17/CXCL8/IL6/RARRES2/SMOC2	7
Air and noise pollution	BP	GO:0071674	mononuclear cell migration	10/102	206/18888	0.0485	8.9891	0.0004	0.0000	CCL11/CCL16/CCL3/CX3CL1/CXCL13/CXCL17/IL6/RARRES2/SLAMF8/TNFRSF11A	10
Air and noise pollution	BP	GO:0030595	leukocyte chemotaxis	11/102	237/18888	0.0464	8.5947	0.0001	0.0000	CCL11/CCL16/CCL3/CX3CL1/CXCL13/CXCL17/CXCL8/IL6/RARRES2/SLAMF8/TNFRSF11A	11
Air and noise pollution	BP	GO:0050729	positive regulation of inflammatory response	7/102	153/18888	0.0458	8.4721	0.0419	0.0008	CCL3/CX3CL1/IL1RL1/IL6/LILRA5/OSMR/TNFRSF11A	7
Air and noise pollution	BP	GO:0045766	positive regulation of angiogenesis	8/102	187/18888	0.0428	7.9220	0.0171	0.0005	ACVRL1/CCL11/CTSH/CXCL8/DDAH1/ENG/HYAL1/SMOC2	8
Air and noise pollution	BP	GO:0048771	tissue remodeling	8/102	188/18888	0.0426	7.8798	0.0177	0.0005	ACVRL1/CD38/EPHA2/IL6/SFRP1/TIMP1/TNFRSF11A/TPP1	8
Air and noise pollution	BP	GO:1904018	positive regulation of vasculature development	8/102	190/18888	0.0421	7.7969	0.0192	0.0005	ACVRL1/CCL11/CTSH/CXCL8/DDAH1/ENG/HYAL1/SMOC2	8
Air and noise pollution	BP	GO:0034612	response to tumor necrosis factor	11/102	263/18888	0.0418	7.7450	0.0004	0.0000	ADAM9/ADAMTS13/CCL11/CCL16/CCL3/CX3CL1/CXCL8/HYAL1/KRT18/SFRP1/TNFRSF11A	11

Supplementary Table 3 GO Enrichment Analysis Results for the Exposure Pattern Proteomic Signature, assessed using a one-sided Fisher's exact test with Bonferroni correction for multiple testing

Expposure pattern	ONTOLOGY	ID	Description	GeneRatio	BgRatio	RichFactor	FoldEnrichment	p.adjust	qvalue	geneID	Count
Air and noise pollution	BP	GO:0071356	cellular response to tumor necrosis factor	10/102	241/18888	0.0415	7.6837	0.0016	0.0001	ADAMTS13/CCL11/CCL16/CCL3/CX3CL1/CXCL8/HYAL1/KRT18/SFRP1/TNFRSF11A	10
Air and noise pollution	BP	GO:0060326	cell chemotaxis	13/102	322/18888	0.0404	7.4761	0.0000	0.0000	CCL11/CCL16/CCL3/CX3CL1/CXCL13/CXCL17/CXCL8/EPHA2/IL6/RARRES2/SLAMF8/SMOC2/TNFRSF11A	13
Air and noise pollution	BP	GO:0050920	regulation of chemotaxis	9/102	229/18888	0.0393	7.2777	0.0090	0.0003	CCL3/CXCL13/CXCL17/CXCL8/IL6/RARRES2/SEMA3F/SLAMF8/SMOC2	9
Air and noise pollution	BP	GO:0002685	regulation of leukocyte migration	9/102	230/18888	0.0391	7.2460	0.0093	0.0003	CCL3/CX3CL1/CXCL13/CXCL17/CXCL8/IL1R1/IL6/RARRES2/SLAMF8	9
Air and noise pollution	BP	GO:1901342	regulation of vasculature development	13/102	362/18888	0.0359	6.6500	0.0002	0.0000	ACVRL1/ANGPTL7/CCL11/CTSH/CXCL13/CXCL8/DDAH1/ENG/EPHA2/HYAL1/IL6/SFRP1/SMOC2	13
Air and noise pollution	BP	GO:0045765	regulation of angiogenesis	12/102	353/18888	0.0340	6.2950	0.0010	0.0000	ACVRL1/CCL11/CTSH/CXCL13/CXCL8/DDAH1/ENG/EPHA2/HYAL1/IL6/SFRP1/SMOC2	12
Air and noise pollution	BP	GO:0006935	chemotaxis	15/102	466/18888	0.0322	5.9606	0.0001	0.0000	CCL11/CCL16/CCL3/CX3CL1/CXCL13/CXCL17/CXCL8/EPHA2/IL6/ITGAV/RARRES2/SEMA3F/SLAMF8/SMOC2/TNFRSF11A	15
Air and noise pollution	BP	GO:0042330	taxis	15/102	468/18888	0.0321	5.9351	0.0001	0.0000	CCL11/CCL16/CCL3/CX3CL1/CXCL13/CXCL17/CXCL8/EPHA2/IL6/ITGAV/RARRES2/SEMA3F/SLAMF8/SMOC2/TNFRSF11A	15
Air and noise pollution	BP	GO:0050900	leukocyte migration	12/102	396/18888	0.0303	5.6114	0.0033	0.0001	CCL11/CCL16/CCL3/CX3CL1/CXCL13/CXCL17/CXCL8/IL1R1/IL6/RARRES2/SLAMF8/TNFRSF11A	12
Air and noise pollution	BP	GO:0070371	ERK1 and ERK2 cascade	10/102	332/18888	0.0301	5.5776	0.0271	0.0006	CCL11/CCL16/CCL3/CTSH/CX3CL1/CXCL17/EPHA2/FGF21/FGF23/ITGAV	10

Supplementary Table 3 GO Enrichment Analysis Results for the Exposure Pattern Proteomic Signature, assessed using a one-sided Fisher's exact test with Bonferroni correction for multiple testing

Expposure pattern	ONTOLOGY	ID	Description	GeneRatio	BgRatio	RichFactor	FoldEnrichment	p.adjust	qvalue	geneID	Count
Air and noise pollution	BP	GO:0043410	positive regulation of MAPK cascade	14/102	475/18888	0.0295	5.4578	0.0006	0.0000	ADAM9/CCL11/CCL16/CCL3/CX3CL1/CXCL17/FGF21/FGF23/GDF15/GPR37/IGFBP4/IL6/LILRA5/TNFRSF11A	14
Air and noise pollution	BP	GO:0032496	response to lipopolysaccharide	10/102	348/18888	0.0287	5.3212	0.0407	0.0008	ADAM9/ADAMTS13/CX3CL1/CXCL13/CXCL8/IL6/REN/SOD2/SPON2/TNFRSF11A	10
Air and noise pollution	BP	GO:0001667	ameboidal-type cell migration	13/102	498/18888	0.0261	4.8339	0.0061	0.0002	ACVRL1/ADAM9/CTSH/CXCL13/EPHA2/FGFBP1/FOLR1/FSTL1/HYAL1/SEMA3F/SMOC2/STC1/TIMP1	13
Air and noise pollution	CC	GO:0005604	basement membrane	5/104	92/19894	0.0543	10.3961	0.0193	0.0037	AGRN/LAMA4/SMOC1/SMOC2/TIMP1	5
Air and noise pollution	CC	GO:0043202	lysosomal lumen	5/104	98/19894	0.0510	9.7596	0.0260	0.0039	AGRN/BCAN/CTSD/HYAL1/TPP	5
Air and noise pollution	CC	GO:0062023	collagen-containing extracellular matrix	16/104	428/19894	0.0374	7.1510	0.0000	0.0000	AGRN/AMBP/ANGPTL7/BCAN/CTSD/CTSH/GDF15/LAMA4/MFGE8/RARRES2/SERPING1/SFRP1/SMOC1/SMOC2/TIMP1/TNR	16
Air and noise pollution	CC	GO:0070820	tertiary granule	6/104	164/19894	0.0366	6.9984	0.0355	0.0039	CD93/CTSD/CTSH/FCAR/NFASC/QPCT	6
Air and noise pollution	CC	GO:0009897	external side of plasma membrane	13/104	405/19894	0.0321	6.1401	0.0000	0.0000	ADAM9/ALCAM/BTN3A2/ENG/FCRLB/FOLR1/GFRA1/IL1R1/IL1RL1/ITGAV/MFGE8/OSMR/TNFRSF11A	13
Air and noise pollution	CC	GO:0005788	endoplasmic reticulum lumen	9/104	313/19894	0.0288	5.5003	0.0062	0.0016	ADAMTS13/FGF23/FSTL1/IGFBP1/IGFBP4/IL6/MFGE8/SERPING1/TIMP1	9
Air and noise pollution	CC	GO:0034774	secretory granule lumen	8/104	322/19894	0.0248	4.7525	0.0460	0.0039	ADA2/CTSD/CTSH/FAM3C/QPCT/RARRES2/SERPING1/TIMP1	8
Air and noise pollution	CC	GO:0060205	cytoplasmic vesicle lumen	8/104	325/19894	0.0246	4.7086	0.0490	0.0039	ADA2/CTSD/CTSH/FAM3C/QPCT/RARRES2/SERPING1/TIMP1	8
Air and noise pollution	CC	GO:0031983	vesicle lumen	8/104	326/19894	0.0245	4.6942	0.0500	0.0039	ADA2/CTSD/CTSH/FAM3C/QPCT/RARRES2/SERPING1/TIMP1	8

Supplementary Table 3 GO Enrichment Analysis Results for the Exposure Pattern Proteomic Signature, assessed using a one-sided Fisher's exact test with Bonferroni correction for multiple testing

Expposure pattern	ONTOLOGY	ID	Description	GeneRatio	BgRatio	RichFactor	FoldEnrichment	p.adjust	qvalue	geneID	Count
Air and noise pollution	MF	GO:0031994	insulin-like growth factor I binding	4/103	13/18522	0.3077	55.3308	0.0001	0.0000	IGFBP1/IGFBP2/IGFBP4/ITGAV	4
Air and noise pollution	MF	GO:0050135	NADP+ nucleosidase activity	4/103	16/18522	0.2500	44.9563	0.0003	0.0000	CD38/IL18R1/IL1R1/IL1RL1	4
Air and noise pollution	MF	GO:0061809	NAD+ nucleotidase, cyclic ADP-ribose generating	4/103	16/18522	0.2500	44.9563	0.0003	0.0000	CD38/IL18R1/IL1R1/IL1RL1	4
Air and noise pollution	MF	GO:0045236	CXCR chemokine receptor binding	4/103	18/18522	0.2222	39.9612	0.0006	0.0000	CX3CL1/CXCL13/CXCL8/TFF2	4
Air and noise pollution	MF	GO:0005520	insulin-like growth factor binding	4/103	19/18522	0.2105	37.8579	0.0007	0.0000	IGFBP1/IGFBP2/IGFBP4/ITGAV	4
Air and noise pollution	MF	GO:0008009	chemokine activity	6/103	49/18522	0.1224	22.0194	0.0001	0.0000	CCL11/CCL16/CCL3/CX3CL1/CXCL13/CXCL8	6
Air and noise pollution	MF	GO:0016799	hydrolase activity, hydrolyzing N-glycosyl compounds	4/103	39/18522	0.1026	18.4436	0.0135	0.0005	CD38/IL18R1/IL1R1/IL1RL1	4
Air and noise pollution	MF	GO:0048020	CCR chemokine receptor binding	5/103	50/18522	0.1000	17.9825	0.0018	0.0001	CCL11/CCL16/CCL3/CX3CL1/CXCL13	5
Air and noise pollution	MF	GO:0042379	chemokine receptor binding	7/103	74/18522	0.0946	17.0105	0.0000	0.0000	CCL11/CCL16/CCL3/CX3CL1/CXCL13/CXCL8/TFF2	7
Air and noise pollution	MF	GO:0050840	extracellular matrix binding	6/103	64/18522	0.0938	16.8586	0.0003	0.0000	ADAM9/ADAMTSL2/AGRN/ITGAV/SMOC1/SMOC2	6
Air and noise pollution	MF	GO:0019838	growth factor binding	11/103	135/18522	0.0815	14.6524	0.0000	0.0000	ACVRL1/CXCL13/ENG/EPHA2/FGFBP1/IGFBP1/IGFBP2/IGFBP4/IL1R1/ITGAV/OSMR	11
Air and noise pollution	MF	GO:0004896	cytokine receptor activity	6/103	96/18522	0.0625	11.2391	0.0033	0.0001	GFRA1/IL10RB/IL18R1/IL1R1/IL1RL1/OSMR	6
Air and noise pollution	MF	GO:0019955	cytokine binding	8/103	145/18522	0.0552	9.9214	0.0003	0.0000	ACVRL1/ENG/IL18R1/IL1R1/IL1RL1/ITGAV/OSMR/TNFRSF11A	8
Air and noise pollution	MF	GO:0140375	immune receptor activity	8/103	151/18522	0.0530	9.5272	0.0004	0.0000	CTSH/GFRA1/IL10RB/IL18R1/IL1R1/IL1RL1/LILRA5/OSMR	8
Air and noise pollution	MF	GO:0005125	cytokine activity	12/103	238/18522	0.0504	9.0668	0.0000	0.0000	CCL11/CCL16/CCL3/CX3CL1/CXCL13/CXCL8/FAM3B/FAM3C/GDF15/IL17C/IL6/TIMP1	12
Air and noise pollution	MF	GO:0005539	glycosaminoglycan binding	11/103	239/18522	0.0460	8.2765	0.0000	0.0000	ADA2/AGRN/BCAN/CXCL13/CXCL8/ENG/FGFBP1/FSTL1/SFRP1/SMOC1/SMOC2	11
Air and noise pollution	MF	GO:0008201	heparin binding	8/103	174/18522	0.0460	8.2678	0.0012	0.0001	ADA2/CXCL13/CXCL8/FGFBP1/FSTL1/SFRP1/SMOC1/SMOC2	8

Supplementary Table 3 GO Enrichment Analysis Results for the Exposure Pattern Proteomic Signature, assessed using a one-sided Fisher's exact test with Bonferroni correction for multiple testing

Expposure pattern	ONTOLOGY	ID	Description	GeneRatio	BgRatio	RichFactor	FoldEnrichment	p.adjust	qvalue	geneID	Count
Air and noise pollution	MF	GO:0008083	growth factor activity	7/103	162/18522	0.0432	7.7702	0.0071	0.0003	ADA2/FGF21/FGF23/GDF15/IL6/TFF1/TIMP1	7
Air and noise pollution	MF	GO:0005126	cytokine receptor binding	10/103	273/18522	0.0366	6.5870	0.0006	0.0000	CCL11/CCL16/CCL3/CX3CL1/CXCL13/CXCL8/ENG/IL6/OSMR/TFF2	10
Air and noise pollution	MF	GO:0001664	G protein-coupled receptor binding	10/103	291/18522	0.0344	6.1796	0.0011	0.0001	ADA2/CCL11/CCL16/CCL3/CX3CL1/CXCL13/CXCL8/GAL/SFRP1/TFF2	10
Air and noise pollution	MF	GO:1901681	sulfur compound binding	9/103	267/18522	0.0337	6.0615	0.0038	0.0002	ADA2/AGRN/CXCL13/CXCL8/FGFBP1/FSTL1/SFRP1/SMOC1/SMOC2	9
Air and noise pollution	MF	GO:0030246	carbohydrate binding	9/103	279/18522	0.0323	5.8008	0.0054	0.0002	AMBP/BCAN/CD93/ENG/FAM3B/FAM3C/GALNT10/SIGLEC1/SIGLEC7	9
Blue and green spaces	BP	GO:0048245	eosinophil chemotaxis	6/145	27/18888	0.2222	28.9471	0.0001	0.0000	CCL11/CCL16/CCL22/CCL3/CX3CL1/SCG2	6
Blue and green spaces	BP	GO:0072677	eosinophil migration	6/145	31/18888	0.1935	25.2120	0.0003	0.0000	CCL11/CCL16/CCL22/CCL3/CX3CL1/SCG2	6
Blue and green spaces	BP	GO:0002548	monocyte chemotaxis	9/145	70/18888	0.1286	16.7480	0.0000	0.0000	CCL11/CCL16/CCL22/CCL3/CX3CL1/CXCL17/IL6/SLAMF8/TNFRSF11A	9
Blue and green spaces	BP	GO:0070555	response to interleukin-1	15/145	138/18888	0.1087	14.1589	0.0000	0.0000	CCL11/CCL16/CCL22/CCL3/CD38/CX3CL1/CXCL8/EDN1/HYAL1/IL1R1/IL6/SELE/SFRP1/SMPD1/TNFRSF11A	15
Blue and green spaces	BP	GO:0071347	cellular response to interleukin-1	11/145	110/18888	0.1000	13.0262	0.0000	0.0000	CCL11/CCL16/CCL22/CCL3/CX3CL1/CXCL8/EDN1/HYAL1/IL1R1/IL6/SFRP1	11
Blue and green spaces	BP	GO:0048247	lymphocyte chemotaxis	6/145	64/18888	0.0938	12.2121	0.0267	0.0004	CCL11/CCL16/CCL22/CCL3/CX3CL1/CXCL13	6
Blue and green spaces	BP	GO:0070098	chemokine-mediated signaling pathway	8/145	93/18888	0.0860	11.2053	0.0016	0.0000	CCL11/CCL16/CCL22/CCL3/CX3CL1/CXCL13/CXCL8/EDN1	8

Supplementary Table 3 GO Enrichment Analysis Results for the Exposure Pattern Proteomic Signature, assessed using a one-sided Fisher's exact test with Bonferroni correction for multiple testing

Expposure pattern	ONTOLOGY	ID	Description	GeneRatio	BgRatio	RichFactor	FoldEnrichment	p.adjust	qvalue	geneID	Count
Blue and green spaces	BP	GO:0071621	granulocyte chemotaxis	11/145	128/18888	0.0859	11.1944	0.0000	0.0000	CCL11/CCL16/CCL22/CCL3/CX3CL1/CXCL13/CXCL17/CXCL8/EDN1/RARRES2/SCG2	11
Blue and green spaces	BP	GO:0050918	positive chemotaxis	6/145	70/18888	0.0857	11.1653	0.0450	0.0006	ANGPT2/CCL16/CCL3/CX3CL1/CXCL8/SCG2	6
Blue and green spaces	BP	GO:0097530	granulocyte migration	13/145	154/18888	0.0844	10.9961	0.0000	0.0000	CCL11/CCL16/CCL22/CCL3/CX3CL1/CXCL13/CXCL17/CXCL8/EDN1/IL1R1/RARRES2/SCG2/SLAMF8	13
Blue and green spaces	BP	GO:1990868	response to chemokine	8/145	101/18888	0.0792	10.3178	0.0031	0.0001	CCL11/CCL16/CCL22/CCL3/CX3CL1/CXCL13/CXCL8/EDN1	8
Blue and green spaces	BP	GO:1990869	cellular response to chemokine	8/145	101/18888	0.0792	10.3178	0.0031	0.0001	CCL11/CCL16/CCL22/CCL3/CX3CL1/CXCL13/CXCL8/EDN1	8
Blue and green spaces	BP	GO:0046849	bone remodeling	7/145	90/18888	0.0778	10.1315	0.0166	0.0003	CD38/EPHA2/IL6/LTBP3/SFRP1/TNFRSF11A/TPP1	7
Blue and green spaces	BP	GO:1990266	neutrophil migration	10/145	129/18888	0.0775	10.0978	0.0002	0.0000	CCL11/CCL16/CCL22/CCL3/CX3CL1/CXCL13/CXCL8/EDN1/IL1R1/SLAMF8	10
Blue and green spaces	BP	GO:0002690	positive regulation of leukocyte chemotaxis	7/145	93/18888	0.0753	9.8047	0.0207	0.0003	CCL3/CXCL13/CXCL17/CXCL8/EDN1/IL6/RARRES2	7
Blue and green spaces	BP	GO:0071346	cellular response to type II interferon	9/145	120/18888	0.0750	9.7697	0.0010	0.0000	ADAMTS13/CCL11/CCL16/CCL22/CCL3/CX3CL1/EDN1/IFNGR1/MRC1	9
Blue and green spaces	BP	GO:0030593	neutrophil chemotaxis	8/145	107/18888	0.0748	9.7392	0.0048	0.0001	CCL11/CCL16/CCL22/CCL3/CX3CL1/CXCL13/CXCL8/EDN1	8
Blue and green spaces	BP	GO:0002687	positive regulation of leukocyte migration	11/145	149/18888	0.0738	9.6167	0.0001	0.0000	CCL3/CX3CL1/CXCL13/CXCL17/CXCL8/EDN1/IL1R1/IL6/KITLG/RARRES2/SELE	11
Blue and green spaces	BP	GO:0032760	positive regulation of tumor necrosis factor production	8/145	109/18888	0.0734	9.5605	0.0055	0.0001	CCL3/CLEC7A/HAVCR2/IFNGR1/IL6/LILRA5/ORM1/SPON2	8
Blue and green spaces	BP	GO:0042116	macrophage activation	8/145	110/18888	0.0727	9.4736	0.0059	0.0001	CCL3/CD93/CX3CL1/GRN/HAVCR2/IFNGR1/IL1RL1/IL6	8
Blue and green spaces	BP	GO:1903557	positive regulation of tumor necrosis factor superfamily cytokine production	8/145	113/18888	0.0708	9.2221	0.0072	0.0001	CCL3/CLEC7A/HAVCR2/IFNGR1/IL6/LILRA5/ORM1/SPON2	8

Supplementary Table 3 GO Enrichment Analysis Results for the Exposure Pattern Proteomic Signature, assessed using a one-sided Fisher's exact test with Bonferroni correction for multiple testing

Expposure pattern	ONTOLOGY	ID	Description	GeneRatio	BgRatio	RichFactor	FoldEnrichment	p.adjust	qvalue	geneID	Count
Blue and green spaces	BP	GO:0034341	response to type II interferon	10/145	142/18888	0.0704	9.1734	0.0004	0.0000	ADAMTS13/BST2/CCL11/CCL16/CCL22/CCL3/CX3CL1/EDN1/IFNGR1/MRC1	10
Blue and green spaces	BP	GO:0044344	cellular response to fibroblast growth factor stimulus	8/145	116/18888	0.0690	8.9836	0.0087	0.0001	CXCL13/CXCL8/FGF21/FGF23/FGFBP1/HYAL1/SFRP1/SMOC2	8
Blue and green spaces	BP	GO:0097529	myeloid leukocyte migration	16/145	239/18888	0.0669	8.7205	0.0000	0.0000	CCL11/CCL16/CCL22/CCL3/CX3CL1/CXCL13/CXCL17/CXCL8/EDN1/IL1R1/IL6/KITLG/RARRES2/SCG2/SLAMF8/TNFRSF11A	16
Blue and green spaces	BP	GO:0002688	regulation of leukocyte chemotaxis	8/145	123/18888	0.0650	8.4723	0.0136	0.0002	CCL3/CXCL13/CXCL17/CXCL8/EDN1/IL6/RARRES2/SLAMF8	8
Blue and green spaces	BP	GO:0071774	response to fibroblast growth factor	8/145	124/18888	0.0645	8.4040	0.0144	0.0002	CXCL13/CXCL8/FGF21/FGF23/FGFBP1/HYAL1/SFRP1/SMOC2	8
Blue and green spaces	BP	GO:0050921	positive regulation of chemotaxis	9/145	142/18888	0.0634	8.2560	0.0042	0.0001	CCL3/CXCL13/CXCL17/CXCL8/EDN1/IL6/RARRES2/SCG2/SMOC2	9
Blue and green spaces	BP	GO:0034612	response to tumor necrosis factor	16/145	263/18888	0.0608	7.9247	0.0000	0.0000	ADAM9/ADAMTS13/CCL11/CCL16/CCL22/CCL3/CX3CL1/CXCL8/EDN1/HYAL1/KRT18/SELE/SFRP1/SMPD1/TNFRSF11A/TNFSF13B	16
Blue and green spaces	BP	GO:0030595	leukocyte chemotaxis	14/145	237/18888	0.0591	7.6948	0.0000	0.0000	CCL11/CCL16/CCL22/CCL3/CX3CL1/CXCL13/CXCL17/CXCL8/EDN1/IL6/RARRES2/SCG2/SLAMF8/TNFRSF11A	14
Blue and green spaces	BP	GO:0050920	regulation of chemotaxis	13/145	229/18888	0.0568	7.3948	0.0001	0.0000	ANGPT2/CCL3/CXCL13/CXCL17/CXCL8/EDN1/IL6/RARRES2/SCG2/SEMA3F/SLAMF8/SMOC2/ST6GAL1	13
Blue and green spaces	BP	GO:0071356	cellular response to tumor necrosis factor	13/145	241/18888	0.0539	7.0266	0.0001	0.0000	ADAMTS13/CCL11/CCL16/CCL22/CCL3/CX3CL1/CXCL8/EDN1/HYAL1/KRT18/SFRP1/TNFRSF11A/TNFSF13B	13

Supplementary Table 3 GO Enrichment Analysis Results for the Exposure Pattern Proteomic Signature, assessed using a one-sided Fisher's exact test with Bonferroni correction for multiple testing

Expposure pattern	ONTOLOGY	ID	Description	GeneRatio	BgRatio	RichFactor	FoldEnrichment	p.adjust	qvalue	geneID	Count
Blue and green spaces	BP	GO:0045766	positive regulation of angiogenesis	10/145	187/18888	0.0535	6.9659	0.0051	0.0001	ACVRL1/ANGPT2/CCL11/CTSH/CXCL8/DDAH1/ENG/GRN/HYAL1/SMOC2	10
Blue and green spaces	BP	GO:0071674	mononuclear cell migration	11/145	206/18888	0.0534	6.9557	0.0016	0.0000	CCL11/CCL16/CCL22/CCL3/CX3CL1/CXCL13/CXCL17/IL6/RARRES2/SLAMF8/TNFRSF11A	11
Blue and green spaces	BP	GO:1904018	positive regulation of vasculature development	10/145	190/18888	0.0526	6.8559	0.0059	0.0001	ACVRL1/ANGPT2/CCL11/CTSH/CXCL8/DDAH1/ENG/GRN/HYAL1/SMOC2	10
Blue and green spaces	BP	GO:0002685	regulation of leukocyte migration	12/145	230/18888	0.0522	6.7963	0.0006	0.0000	CCL3/CX3CL1/CXCL13/CXCL17/CXCL8/EDN1/IL1R1/IL6/KITLG/RARRES2/SELE/SLAMF8	12
Blue and green spaces	BP	GO:0060326	cell chemotaxis	16/145	322/18888	0.0497	6.4726	0.0000	0.0000	CCL11/CCL16/CCL22/CCL3/CX3CL1/CXCL13/CXCL17/CXCL8/EDN1/EPHA2/IL6/RARRES2/SCG2/SLAMF8/SMOC2/TNFRSF11A	16
Blue and green spaces	BP	GO:0071216	cellular response to biotic stimulus	13/145	265/18888	0.0491	6.3902	0.0004	0.0000	ADAM9/ADAMTS13/CLEC7A/CX3CL1/CXCL13/CXCL8/HAVCR2/IGFBPL1/IL6/MMP9/MRC1/SPON2/TIGAR	13
Blue and green spaces	BP	GO:1905952	regulation of lipid localization	9/145	186/18888	0.0484	6.3030	0.0384	0.0005	ABCA2/EDN1/ENPP7/FABP3/GAL/IL6/ITGAV/REN/TNFRSF11A	9
Blue and green spaces	BP	GO:0048771	tissue remodeling	9/145	188/18888	0.0479	6.2360	0.0418	0.0006	ACVRL1/CD38/EPHA2/IL6/LTBP3/SFRP1/TIMP1/TNFRSF11A/TPP1	9
Blue and green spaces	BP	GO:0032640	tumor necrosis factor production	9/145	189/18888	0.0476	6.2030	0.0436	0.0006	CCL3/CLEC7A/CX3CL1/HAVCR2/IFNGR1/IL6/LILRA5/ORM1/SPON2	9
Blue and green spaces	BP	GO:0032680	regulation of tumor necrosis factor production	9/145	189/18888	0.0476	6.2030	0.0436	0.0006	CCL3/CLEC7A/CX3CL1/HAVCR2/IFNGR1/IL6/LILRA5/ORM1/SPON2	9
Blue and green spaces	BP	GO:0002274	myeloid leukocyte activation	11/145	241/18888	0.0456	5.9456	0.0073	0.0001	ADAM9/CCL3/CD93/CX3CL1/CXCL8/GRN/HAVCR1/HAVCR2/IFNGR1/IL1RL1/IL6	11

Supplementary Table 3 GO Enrichment Analysis Results for the Exposure Pattern Proteomic Signature, assessed using a one-sided Fisher's exact test with Bonferroni correction for multiple testing

Expposure pattern	ONTOLOGY	ID	Description	GeneRatio	BgRatio	RichFactor	FoldEnrichment	p.adjust	qvalue	geneID	Count
Blue and green spaces	BP	GO:0071222	cellular response to lipopolysaccharide	10/145	225/18888	0.0444	5.7894	0.0267	0.0004	ADAM9/ADAMTS13/CX3CL1/CXCL13/CXCL8/HAVCR2/IL6/MMP9/MRC1/SPON2	10
Blue and green spaces	BP	GO:0010632	regulation of epithelial cell migration	13/145	298/18888	0.0436	5.6826	0.0014	0.0000	ACVRL1/ADAM9/ANGPT2/CTSH/CXCL13/EDN1/EPHA2/FGFBP1/GRN/HYAL1/MMP9/SMOC2/STC1	13
Blue and green spaces	BP	GO:0097191	extrinsic apoptotic signaling pathway	10/145	230/18888	0.0435	5.6636	0.0323	0.0005	CX3CL1/IL19/ITGAV/KITLG/KRT18/SCG2/SFRP1/TNFRSF10A/TNFRSF10C/TNFRSF12A	10
Blue and green spaces	BP	GO:0032496	response to lipopolysaccharide	15/145	348/18888	0.0431	5.6147	0.0002	0.0000	ADAM9/ADAMTS13/CX3CL1/CXCL13/CXCL8/EDN1/HAVCR2/IL6/MMP9/MRC1/REN/SELE/SOD2/SPON2/TNFRSF11A	15
Blue and green spaces	BP	GO:0050900	leukocyte migration	17/145	396/18888	0.0429	5.5921	0.0000	0.0000	CCL11/CCL16/CCL22/CCL3/CX3CL1/CXCL13/CXCL17/CXCL8/EDN1/IL1R1/IL6/KITLG/RARRES2/SCG2/SELE/SLAMF8/TNFRSF11A	17
Blue and green spaces	BP	GO:0006935	chemotaxis	20/145	466/18888	0.0429	5.5906	0.0000	0.0000	ANGPT2/CCL11/CCL16/CCL22/CCL3/CX3CL1/CXCL13/CXCL17/CXCL8/EDN1/EPHA2/IL6/ITGAV/RARRES2/SCG2/SEMA3F/SLAMF8/SMOC2/ST6GAL1/TNFRSF11A	20
Blue and green spaces	BP	GO:0042330	taxis	20/145	468/18888	0.0427	5.5668	0.0000	0.0000	ANGPT2/CCL11/CCL16/CCL22/CCL3/CX3CL1/CXCL13/CXCL17/CXCL8/EDN1/EPHA2/IL6/ITGAV/RARRES2/SCG2/SEMA3F/SLAMF8/SMOC2/ST6GAL1/TNFRSF11A	20
Blue and green spaces	BP	GO:0010631	epithelial cell migration	16/145	375/18888	0.0427	5.5578	0.0001	0.0000	ACVRL1/ADAM9/ANGPT2/CTSH/CXCL13/EDN1/EPHA2/FGFBP1/FSTL1/GRN/HYAL1/KITLG/MMP9/SCG2/SMOC2/STC1	16

Supplementary Table 3 GO Enrichment Analysis Results for the Exposure Pattern Proteomic Signature, assessed using a one-sided Fisher's exact test with Bonferroni correction for multiple testing

Expposure pattern	ONTOLOGY	ID	Description	GeneRatio	BgRatio	RichFactor	FoldEnrichment	p.adjust	qvalue	geneID	Count
Blue and green spaces	BP	GO:0090132	epithelium migration	16/145	378/18888	0.0423	5.5137	0.0001	0.0000	ACVRL1/ADAM9/ANGPT2/CTSH/CXCL13/EDN1/EPHA2/FGFBP1/FSTL1/GRN/HYAL1/KITLG/MMP9/SCG2/SMOC2/STC1	16
Blue and green spaces	BP	GO:0071219	cellular response to molecule of bacterial origin	10/145	238/18888	0.0420	5.4732	0.0434	0.0006	ADAM9/ADAMTS13/CX3CL1/CXCL13/CXCL8/HAVCR2/IL6/MMP9/MRC1/SPON2	10
Blue and green spaces	BP	GO:0090130	tissue migration	16/145	383/18888	0.0418	5.4418	0.0001	0.0000	ACVRL1/ADAM9/ANGPT2/CTSH/CXCL13/EDN1/EPHA2/FGFBP1/FSTL1/GRN/HYAL1/KITLG/MMP9/SCG2/SMOC2/STC1	16
Blue and green spaces	BP	GO:0032944	regulation of mononuclear cell proliferation	10/145	241/18888	0.0415	5.4051	0.0484	0.0006	CD38/GAL/HAVCR2/IGFBP2/IL6/KITLG/MAD1L1/MZB1/ST6GAL1/TNFSF13B	10
Blue and green spaces	BP	GO:1901342	regulation of vasculature development	15/145	362/18888	0.0414	5.3976	0.0004	0.0000	ACVRL1/ANGPT2/ANGPTL7/CCL11/CTSH/CXCL13/CXCL8/DDAH1/ENG/EPHA2/GRN/HYAL1/IL6/SFRP1/SMOC2	15
Blue and green spaces	BP	GO:0070663	regulation of leukocyte proliferation	11/145	267/18888	0.0412	5.3666	0.0194	0.0003	BST2/CD38/GAL/HAVCR2/IGFBP2/IL6/KITLG/MAD1L1/MZB1/ST6GAL1/TNFSF13B	11
Blue and green spaces	BP	GO:0002237	response to molecule of bacterial origin	15/145	369/18888	0.0407	5.2952	0.0005	0.0000	ADAM9/ADAMTS13/CX3CL1/CXCL13/CXCL8/EDN1/HAVCR2/IL6/MMP9/MRC1/REN/SELE/SOD2/SPON2/TNFRSF11A	15
Blue and green spaces	BP	GO:0045765	regulation of angiogenesis	14/145	353/18888	0.0397	5.1662	0.0016	0.0000	ACVRL1/ANGPT2/CCL11/CTSH/CXCL13/CXCL8/DDAH1/ENG/EPHA2/GRN/HYAL1/IL6/SFRP1/SMOC2	14
Blue and green spaces	BP	GO:0070371	ERK1 and ERK2 cascade	13/145	332/18888	0.0392	5.1006	0.0049	0.0001	CCL11/CCL16/CCL22/CCL3/CTSH/CX3CL1/CXCL17/EDN1/EPHA2/FGF21/FGF23/HAVCR2/ITGAV	13

Supplementary Table 3 GO Enrichment Analysis Results for the Exposure Pattern Proteomic Signature, assessed using a one-sided Fisher's exact test with Bonferroni correction for multiple testing

Expposure pattern	ONTOLOGY	ID	Description	GeneRatio	BgRatio	RichFactor	FoldEnrichment	p.adjust	qvalue	geneID	Count
Blue and green spaces	BP	GO:0043542	endothelial cell migration	11/145	286/18888	0.0385	5.0101	0.0371	0.0005	ACVRL1/ANGPT2/CXCL13/EDN1/EPHA2/FGFBP1/FSTL1/GRN/SCG2/SMOC2/STC1	11
Blue and green spaces	BP	GO:0001667	ameboidal-type cell migration	19/145	498/18888	0.0382	4.9698	0.0000	0.0000	ACVRL1/ADAM9/ANGPT2/CTSH/CXCL13/EDN1/EPHA2/FGFBP1/FOLR1/FSTL1/GRN/HYAL1/KITLG/MMP9/SCG2/SEMA3F/SMOC2/STC1/TIMP1	19
Blue and green spaces	BP	GO:0043410	positive regulation of MAPK cascade	18/145	475/18888	0.0379	4.9362	0.0001	0.0000	ADAM9/CCL11/CCL16/CCL22/CCL3/CX3CL1/CXCL17/EDAR/EDN1/FGF21/FGF23/GDF15/GPR37/HAVCR2/IGFBP4/IL6/LILRA5/TNFRSF11A	18
Blue and green spaces	BP	GO:0070661	leukocyte proliferation	12/145	352/18888	0.0341	4.4408	0.0498	0.0006	BST2/CD38/CX3CL1/GAL/HAVCR2/IGFBP2/IL6/KITLG/MAD1L1/MZB1/ST6GAL1/TNFSF13B	12
Blue and green spaces	BP	GO:0001819	positive regulation of cytokine production	15/145	500/18888	0.0300	3.9079	0.0201	0.0003	BTN3A2/CCL3/CLEC7A/CX3CL1/CXCL17/HAVCR2/IFNGR1/IL18R1/IL1R1/IL1RL1/IL6/LILRA5/MMP12/ORM1/SPON2	15
Blue and green spaces	CC	GO:1904724	tertiary granule lumen	5/147	55/19894	0.0909	12.3030	0.0114	0.0015	CTSD/CTSH/MMP9/ORM1/QPCT	5
Blue and green spaces	CC	GO:0043202	lysosomal lumen	7/147	98/19894	0.0714	9.6667	0.0018	0.0003	AGRN/BCAN/CTSD/HYAL1/IFI30/SMPD1/TPP1	7
Blue and green spaces	CC	GO:0062023	collagen-containing extracellular matrix	24/147	428/19894	0.0561	7.5888	0.0000	0.0000	AGRN/AMBP/ANGPT2/ANGPTL7/BCAN/CRELD1/CTSD/CTSH/EGFL7/GDF15/IGFBPL1/LAMA4/LGALS4/LTBP3/MFGE8/MMP9/ORM1/RARRES2/SERPING1/SFRP1/SMOC2/TIMP1/TNR/VWA1	24
Blue and green spaces	CC	GO:0005775	vacuolar lumen	9/147	176/19894	0.0511	6.9205	0.0014	0.0003	ADA2/AGRN/BCAN/CTSD/GRN/HYAL1/IFI30/SMPD1/TPP1	9
Blue and green spaces	CC	GO:0070820	tertiary granule	7/147	164/19894	0.0427	5.7764	0.0472	0.0038	CD93/CTSD/CTSH/FCAR/MMP9/ORM1/QPCT	7

Supplementary Table 3 GO Enrichment Analysis Results for the Exposure Pattern Proteomic Signature, assessed using a one-sided Fisher's exact test with Bonferroni correction for multiple testing

Expposure pattern	ONTOLOGY	ID	Description	GeneRatio	BgRatio	RichFactor	FoldEnrichment	p.adjust	qvalue	geneID	Count
Blue and green spaces	CC	GO:0005788	endoplasmic reticulum lumen	13/147	313/19894	0.0415	5.6209	0.0001	0.0001	ADAMTS13/EDN1/FGF23/FSTL1/IGFBP1/IGFBP4/IL6/MFGE8/MZB1/SCG2/SERPING1/TIMP1/VWA1	13
Blue and green spaces	CC	GO:0009897	external side of plasma membrane	13/147	405/19894	0.0321	4.3440	0.0022	0.0003	ADAM9/ALCAM/BTN3A2/ENG/FOLR1/GFRA1/IL1R1/IL1RL1/ITGAV/MFGE8/OSMR/SELE/TNFRSF11A	13
Blue and green spaces	CC	GO:0034774	secretory granule lumen	10/147	322/19894	0.0311	4.2029	0.0308	0.0030	ADA2/CTSD/CTSH/FAM3C/GRN/ORM1/QPCT/RARRES2/SERPING1/TIMP1	10
Blue and green spaces	CC	GO:0060205	cytoplasmic vesicle lumen	10/147	325/19894	0.0308	4.1641	0.0333	0.0030	ADA2/CTSD/CTSH/FAM3C/GRN/ORM1/QPCT/RARRES2/SERPING1/TIMP1	10
Blue and green spaces	CC	GO:0031983	vesicle lumen	10/147	326/19894	0.0307	4.1513	0.0341	0.0030	ADA2/CTSD/CTSH/FAM3C/GRN/ORM1/QPCT/RARRES2/SERPING1/TIMP1	10
Blue and green spaces	MF	GO:0031994	insulin-like growth factor I binding	4/147	13/18522	0.3077	38.7692	0.0007	0.0000	IGFBP1/IGFBP2/IGFBP4/ITGAV	4
Blue and green spaces	MF	GO:0005520	insulin-like growth factor binding	5/147	19/18522	0.2632	33.1579	0.0001	0.0000	IGFBP1/IGFBP2/IGFBP4/IGFBPL1/ITGAV	5
Blue and green spaces	MF	GO:0050135	NADP+ nucleosidase activity	4/147	16/18522	0.2500	31.5000	0.0016	0.0001	CD38/IL18R1/IL1R1/IL1RL1	4
Blue and green spaces	MF	GO:0061809	NAD+ nucleotidase, cyclic ADP-ribose generating	4/147	16/18522	0.2500	31.5000	0.0016	0.0001	CD38/IL18R1/IL1R1/IL1RL1	4
Blue and green spaces	MF	GO:0050431	transforming growth factor beta binding	4/147	24/18522	0.1667	21.0000	0.0092	0.0004	ACVRL1/ENG/ITGAV/LTBP3	4
Blue and green spaces	MF	GO:0008009	chemokine activity	7/147	49/18522	0.1429	18.0000	0.0000	0.0000	CCL11/CCL16/CCL22/CCL3/CX3CL1/CXCL13/CXCL8	7
Blue and green spaces	MF	GO:0048020	CCR chemokine receptor binding	6/147	50/18522	0.1200	15.1200	0.0007	0.0000	CCL11/CCL16/CCL22/CCL3/CX3CL1/CXCL13	6
Blue and green spaces	MF	GO:0019838	growth factor binding	13/147	135/18522	0.0963	12.1333	0.0000	0.0000	ACVRL1/CXCL13/ENG/EPHA2/FGFBP1/IGFBP1/IGFBP2/IGFBP4/IGFBPL1/IL1R1/ITGAV/LTBP3/OSMR	13

Supplementary Table 3 GO Enrichment Analysis Results for the Exposure Pattern Proteomic Signature, assessed using a one-sided Fisher's exact test with Bonferroni correction for multiple testing

Expposure pattern	ONTOLOGY	ID	Description	GeneRatio	BgRatio	RichFactor	FoldEnrichment	p.adjust	qvalue	geneID	Count
Blue and green spaces	MF	GO:0042379	chemokine receptor binding	7/147	74/18522	0.0946	11.9189	0.0005	0.0000	CCL11/CCL16/CCL22/CCL3/CX3CL1/CXCL13/CXCL8	7
Blue and green spaces	MF	GO:0050840	extracellular matrix binding	6/147	64/18522	0.0938	11.8125	0.0030	0.0001	ADAM9/ADAMTSL2/AGRN/ITGAV/SMOC2/VWA1	6
Blue and green spaces	MF	GO:0005125	cytokine activity	19/147	238/18522	0.0798	10.0588	0.0000	0.0000	CCL11/CCL16/CCL22/CCL3/CX3CL1/CXCL13/CXCL8/EDN1/FAM3B/FAM3C/GDF15/GRN/IL17C/IL19/IL6/KITLG/SCG2/TIMP1/TNFSF13B	19
Blue and green spaces	MF	GO:0004896	cytokine receptor activity	7/147	96/18522	0.0729	9.1875	0.0029	0.0001	GFRA1/IFNGR1/IL10RB/IL18R1/IL1R1/IL1RL1/OSMR	7
Blue and green spaces	MF	GO:0019955	cytokine binding	10/147	145/18522	0.0690	8.6897	0.0001	0.0000	ACVRL1/ENG/IFNGR1/IL18R1/IL1R1/IL1RL1/ITGAV/LTBP3/OSMR/TNFRSF11A	10
Blue and green spaces	MF	GO:0140375	immune receptor activity	10/147	151/18522	0.0662	8.3444	0.0001	0.0000	CTSH/GFRA1/IFNGR1/IL10RB/IL18R1/IL1R1/IL1RL1/LILRA5/OSMR/PIGR	10
Blue and green spaces	MF	GO:0008083	growth factor activity	9/147	162/18522	0.0556	7.0000	0.0015	0.0001	ADA2/FGF21/FGF23/GDF15/GRN/IL6/KITLG/TFF1/TIMP1	9
Blue and green spaces	MF	GO:0030246	carbohydrate binding	15/147	279/18522	0.0538	6.7742	0.0000	0.0000	AMBP/BCAN/CD93/CLEC7A/ENG/FAM3B/FAM3C/GALNT10/KLRB1/LGALS4/MRC1/SELE/SFTPA2/SIGLEC1/SIGLEC7	15
Blue and green spaces	MF	GO:0005126	cytokine receptor binding	12/147	273/18522	0.0440	5.5385	0.0005	0.0000	CCL11/CCL16/CCL22/CCL3/CX3CL1/CXCL13/CXCL8/ENG/IL6/KITLG/OSMR/TNFSF13B	12
Blue and green spaces	MF	GO:0005539	glycosaminoglycan binding	10/147	239/18522	0.0418	5.2720	0.0055	0.0002	ADA2/AGRN/BCAN/CXCL13/CXCL8/ENG/FGFBP1/FSTL1/SFRP1/SMOC2	10
Blue and green spaces	MF	GO:0031406	carboxylic acid binding	8/147	196/18522	0.0408	5.1429	0.0444	0.0016	AGRN/AMBP/DDAH1/FABP3/FOLR1/RBP2/SELE/SIGLEC7	8

Supplementary Table 3 GO Enrichment Analysis Results for the Exposure Pattern Proteomic Signature, assessed using a one-sided Fisher's exact test with Bonferroni correction for multiple testing

Expposure pattern	ONTOLOGY	ID	Description	GeneRatio	BgRatio	RichFactor	FoldEnrichment	p.adjust	qvalue	geneID	Count
Blue and green spaces	MF	GO:0001664	G protein-coupled receptor binding	11/147	291/18522	0.0378	4.7629	0.0054	0.0002	ADA2/CCL11/CCL16/CCL22/CCL3/CX3CL1/CXCL13/CXCL8/EDN1/GAL/SFRP1	11
Blue and green spaces	MF	GO:0004175	endopeptidase activity	12/147	388/18522	0.0309	3.8969	0.0163	0.0006	ADAM9/ADAMTS13/CTSD/CTSH/CTSO/KLK8/MME/MMP12/MMP9/REN/SFRP1/TPP1	12
Health behaviors	BP	GO:1905523	positive regulation of macrophage migration	5/154	25/18888	0.2000	24.5299	0.0042	0.0002	CCL3/CX3CL1/CXCL17/RARRES2/TREM2	5
Health behaviors	BP	GO:0043171	peptide catabolic process	5/154	33/18888	0.1515	18.5832	0.0180	0.0005	ADAMTS13/CTSH/GGT1/MME/TPP1	5
Health behaviors	BP	GO:1905521	regulation of macrophage migration	6/154	44/18888	0.1364	16.7249	0.0039	0.0001	CCL3/CX3CL1/CXCL17/RARRES2/SLAMF8/TREM2	6
Health behaviors	BP	GO:0002548	monocyte chemotaxis	9/154	70/18888	0.1286	15.7692	0.0000	0.0000	CCL11/CCL16/CCL20/CCL3/CX3CL1/CXCL17/NBL1/SLAMF8/TNFRSF11A	9
Health behaviors	BP	GO:1905517	macrophage migration	6/154	60/18888	0.1000	12.2649	0.0250	0.0006	CCL3/CX3CL1/CXCL17/RARRES2/SLAMF8/TREM2	6
Health behaviors	BP	GO:0048247	lymphocyte chemotaxis	6/154	64/18888	0.0938	11.4984	0.0364	0.0008	CCL11/CCL16/CCL20/CCL3/CX3CL1/CXCL13	6
Health behaviors	BP	GO:0070555	response to interleukin-1	11/154	138/18888	0.0797	9.7764	0.0000	0.0000	CCL11/CCL16/CCL20/CCL3/CD38/CHI3L1/CX3CL1/HYAL1/IL1R1/SFRP1/TNFRSF11A	11
Health behaviors	BP	GO:0046849	bone remodeling	7/154	90/18888	0.0778	9.5394	0.0239	0.0006	ACP5/CD38/EPHA2/PTN/SFRP1/TNFRSF11A/TPP1	7
Health behaviors	BP	GO:0070098	chemokine-mediated signaling pathway	7/154	93/18888	0.0753	9.2317	0.0297	0.0007	CCL11/CCL16/CCL20/CCL3/CX3CL1/CXCL13/TREM2	7
Health behaviors	BP	GO:1901890	positive regulation of cell junction assembly	8/154	108/18888	0.0741	9.0851	0.0077	0.0003	ACVRL1/AGRN/CLSTN2/EPHA2/SFRP1/SLITRK1/THBS2/THY1	8
Health behaviors	BP	GO:0071347	cellular response to interleukin-1	8/154	110/18888	0.0727	8.9200	0.0089	0.0003	CCL11/CCL16/CCL20/CCL3/CX3CL1/HYAL1/IL1R1/SFRP1	8
Health behaviors	BP	GO:0002687	positive regulation of leukocyte migration	10/154	149/18888	0.0671	8.2315	0.0011	0.0001	CCL20/CCL3/CX3CL1/CXCL13/CXCL17/IL1R1/PTN/RARRES2/THY1/TREM2	10

Supplementary Table 3 GO Enrichment Analysis Results for the Exposure Pattern Proteomic Signature, assessed using a one-sided Fisher's exact test with Bonferroni correction for multiple testing

Expposure pattern	ONTOLOGY	ID	Description	GeneRatio	BgRatio	RichFactor	FoldEnrichment	p.adjust	qvalue	geneID	Count
Health behaviors	BP	GO:0097530	granulocyte migration	10/154	154/18888	0.0649	7.9642	0.0015	0.0001	CCL11/CCL16/CCL20/CCL3/CX3CL1/CXCL13/CXCL17/IL1R1/RARRES2/SLAMF8	10
Health behaviors	BP	GO:0045766	positive regulation of angiogenesis	12/154	187/18888	0.0642	7.8705	0.0001	0.0000	ACVRL1/ADM/BMPER/CCL11/CHI3L1/CTSH/DDAH1/ENG/HSPB6/HYAL1/SMOC2/TGFBR2	12
Health behaviors	BP	GO:1904018	positive regulation of vasculature development	12/154	190/18888	0.0632	7.7463	0.0001	0.0000	ACVRL1/ADM/BMPER/CCL11/CHI3L1/CTSH/DDAH1/ENG/HSPB6/HYAL1/SMOC2/TGFBR2	12
Health behaviors	BP	GO:0071621	granulocyte chemotaxis	8/154	128/18888	0.0625	7.6656	0.0274	0.0007	CCL11/CCL16/CCL20/CCL3/CX3CL1/CXCL13/CXCL17/RARRES2	8
Health behaviors	BP	GO:1990266	neutrophil migration	8/154	129/18888	0.0620	7.6062	0.0290	0.0007	CCL11/CCL16/CCL20/CCL3/CX3CL1/CXCL13/IL1R1/SLAMF8	8
Health behaviors	BP	GO:0071356	cellular response to tumor necrosis factor	14/154	241/18888	0.0581	7.1249	0.0000	0.0000	ADAMTS13/CCL11/CCL16/CCL20/CCL3/CHI3L1/CX3CL1/EDA2R/FABP4/HYAL1/KRT18/SFRP1/TNFRSF11A/TNFSF13B	14
Health behaviors	BP	GO:0034612	response to tumor necrosis factor	15/154	263/18888	0.0570	6.9952	0.0000	0.0000	ADAM9/ADAMTS13/CCL11/CCL16/CCL20/CCL3/CHI3L1/CX3CL1/EDA2R/FABP4/HYAL1/KRT18/SFRP1/TNFRSF11A/TNFSF13B	15
Health behaviors	BP	GO:0097529	myeloid leukocyte migration	13/154	239/18888	0.0544	6.6713	0.0002	0.0000	CCL11/CCL16/CCL20/CCL3/CX3CL1/CXCL13/CXCL17/IL1R1/NBL1/RARRES2/SLAMF8/TNFRSF11A/TREM2	13
Health behaviors	BP	GO:0071674	mononuclear cell migration	11/154	206/18888	0.0534	6.5492	0.0028	0.0001	CCL11/CCL16/CCL20/CCL3/CX3CL1/CXCL13/CXCL17/NBL1/RARRES2/SLAMF8/TNFRSF11A	11
Health behaviors	BP	GO:0002685	regulation of leukocyte migration	12/154	230/18888	0.0522	6.3991	0.0011	0.0001	CCL20/CCL3/CX3CL1/CXCL13/CXCL17/IL1R1/NBL1/PTN/RARRES2/SLAMF8/THY1/TREM2	12

Supplementary Table 3 GO Enrichment Analysis Results for the Exposure Pattern Proteomic Signature, assessed using a one-sided Fisher's exact test with Bonferroni correction for multiple testing

Expposure pattern	ONTOLOGY	ID	Description	GeneRatio	BgRatio	RichFactor	FoldEnrichment	p.adjust	qvalue	geneID	Count
Health behaviors	BP	GO:0030595	leukocyte chemotaxis	12/154	237/18888	0.0506	6.2101	0.0015	0.0001	CCL11/CCL16/CCL20/CCL3/CX3CL1/CXCL13/CXCL17/NBL1/PTN/RARRES2/SLAMF8/TNFRSF11A	12
Health behaviors	BP	GO:1901342	regulation of vasculature development	18/154	362/18888	0.0497	6.0986	0.0000	0.0000	ACVRL1/ADM/ANGPTL7/BMPER/CCL11/CHI3L1/CTSH/CXCL13/DDAH1/ENG/EPHA2/HSPB6/HYAL1/SFRP1/SMOC2/TAFA5/TGFBR2/THBS2	18
Health behaviors	BP	GO:0045765	regulation of angiogenesis	17/154	353/18888	0.0482	5.9066	0.0000	0.0000	ACVRL1/ADM/BMPER/CCL11/CHI3L1/CTSH/CXCL13/DDAH1/ENG/EPHA2/HSPB6/HYAL1/SFRP1/SMOC2/TAFA5/TGFBR2/THBS2	17
Health behaviors	BP	GO:0070374	positive regulation of ERK1 and ERK2 cascade	10/154	215/18888	0.0465	5.7046	0.0294	0.0007	BMPER/CCL11/CCL16/CCL20/CCL3/CHI3L1/CX3CL1/CXCL17/FGF23/TREM2	10
Health behaviors	BP	GO:0060326	cell chemotaxis	14/154	322/18888	0.0435	5.3326	0.0011	0.0001	CCL11/CCL16/CCL20/CCL3/CX3CL1/CXCL13/CXCL17/EPHA2/NBL1/PTN/RARRES2/SLAMF8/SMOC2/TNFRSF11A	14
Health behaviors	BP	GO:0010632	regulation of epithelial cell migration	12/154	298/18888	0.0403	4.9389	0.0167	0.0005	ACVRL1/ADAM9/BMPER/CTSH/CXCL13/EPHA2/FGFBP1/HYAL1/PTN/SMOC2/STC1/TGFBR2	12
Health behaviors	BP	GO:0032496	response to lipopolysaccharide	14/154	348/18888	0.0402	4.9342	0.0028	0.0001	ACP5/ADAM9/ADAMTS13/ADM/C2/CX3CL1/CXCL13/MPO/REN/SOD2/SPON2/THBD/TNFRSF11A/TREM2	14
Health behaviors	BP	GO:0070371	ERK1 and ERK2 cascade	13/154	332/18888	0.0392	4.8025	0.0092	0.0003	BMPER/CCL11/CCL16/CCL20/CCL3/CHI3L1/CTSH/CX3CL1/CXCL17/EPHA2/FGF23/ITGAV/TREM2	13
Health behaviors	BP	GO:0002237	response to molecule of bacterial origin	14/154	369/18888	0.0379	4.6534	0.0055	0.0002	ACP5/ADAM9/ADAMTS13/ADM/C2/CX3CL1/CXCL13/MPO/REN/SOD2/SPON2/THBD/TNFRSF11A/TREM2	14

Supplementary Table 3 GO Enrichment Analysis Results for the Exposure Pattern Proteomic Signature, assessed using a one-sided Fisher's exact test with Bonferroni correction for multiple testing

Expposure pattern	ONTOLOGY	ID	Description	GeneRatio	BgRatio	RichFactor	FoldEnrichment	p.adjust	qvalue	geneID	Count
Health behaviors	BP	GO:0043410	positive regulation of MAPK cascade	18/154	475/18888	0.0379	4.6478	0.0002	0.0000	ADAM9/BMPER/CCL11/CCL16/CCL20/CCL3/CD27/CHI3L1/CX3CL1/CXCL17/EDA2R/FGF23/GDF15/GPR37/IGFBP4/LILRA5/TNFRSF11A/TREM2	18
Health behaviors	BP	GO:0050900	leukocyte migration	15/154	396/18888	0.0379	4.6458	0.0024	0.0001	CCL11/CCL16/CCL20/CCL3/CX3CL1/CXCL13/CXCL17/IL1R1/NBL1/PTN/RARRES2/SLAMF8/THY1/TNFRSF11A/TREM2	15
Health behaviors	BP	GO:0006935	chemotaxis	17/154	466/18888	0.0365	4.4743	0.0007	0.0001	CCL11/CCL16/CCL20/CCL3/CX3CL1/CXCL13/CXCL17/EPHA2/ITGAV/NBL1/PTN/RARRES2/SEMA3F/SLAMF8/SMOC2/TNFRSF11A/TREM2	17
Health behaviors	BP	GO:0042330	taxis	17/154	468/18888	0.0363	4.4552	0.0008	0.0001	CCL11/CCL16/CCL20/CCL3/CX3CL1/CXCL13/CXCL17/EPHA2/ITGAV/NBL1/PTN/RARRES2/SEMA3F/SLAMF8/SMOC2/TNFRSF11A/TREM2	17
Health behaviors	BP	GO:0010631	epithelial cell migration	13/154	375/18888	0.0347	4.2518	0.0342	0.0007	ACVRL1/ADAM9/BMPER/CTSH/CXCL13/EPHA2/FGFBP1/FSTL1/HYAL1/PTN/SMOC2/STC1/TGFBR2	13
Health behaviors	BP	GO:0090132	epithelium migration	13/154	378/18888	0.0344	4.2181	0.0372	0.0008	ACVRL1/ADAM9/BMPER/CTSH/CXCL13/EPHA2/FGFBP1/FSTL1/HYAL1/PTN/SMOC2/STC1/TGFBR2	13
Health behaviors	BP	GO:0090130	tissue migration	13/154	383/18888	0.0339	4.1630	0.0427	0.0008	ACVRL1/ADAM9/BMPER/CTSH/CXCL13/EPHA2/FGFBP1/FSTL1/HYAL1/PTN/SMOC2/STC1/TGFBR2	13

Supplementary Table 3 GO Enrichment Analysis Results for the Exposure Pattern Proteomic Signature, assessed using a one-sided Fisher's exact test with Bonferroni correction for multiple testing

Expposure pattern	ONTOLOGY	ID	Description	GeneRatio	BgRatio	RichFactor	FoldEnrichment	p.adjust	qvalue	geneID	Count
Health behaviors	BP	GO:0002460	adaptive immune response based on somatic recombination of immune receptors built from immunoglobulin superfamily domains	13/154	385/18888	0.0338	4.1414	0.0452	0.0009	BTN3A2/C2/CD27/CTSH/CXCL13/IL18R1/IL1R1/IL1RL1/LILRB4/SERPING1/TNFSF13B/TREM2/ULBP2	13
Health behaviors	BP	GO:0001667	ameboidal-type cell migration	16/154	498/18888	0.0321	3.9405	0.0087	0.0003	ACVRL1/ADAM9/BMPER/CTSH/CXCL13/EPHA2/FGFBP1/FOLR1/FSTL1/HYAL1/PTN/SEMA3F/SMOC2/STC1/TGFBR2/TIMP1	16
Health behaviors	BP	GO:0045785	positive regulation of cell adhesion	15/154	485/18888	0.0309	3.7933	0.0283	0.0007	ADAM9/CCDC80/CD27/CEACAM6/CX3CL1/CXCL13/EDIL3/HYAL1/IGFBP2/ITGAV/LILRB4/SFRP1/TGFBR2/THY1/TNFSF13B	15
Health behaviors	CC	GO:0031089	platelet dense granule lumen	3/157	14/19894	0.2143	27.1529	0.0349	0.0023	FAM3C/LGALS3BP/RARRES2	3
Health behaviors	CC	GO:0005604	basement membrane	6/157	92/19894	0.0652	8.2639	0.0190	0.0015	AGRN/CCDC80/LAMA4/SMOC2/THBS2/TIMP1	6
Health behaviors	CC	GO:0043202	lysosomal lumen	6/157	98/19894	0.0612	7.7580	0.0269	0.0020	AGRN/BCAN/CTSD/HYAL1/SDC1/TPP1	6
Health behaviors	CC	GO:0009897	external side of plasma membrane	21/157	405/19894	0.0519	6.5703	0.0000	0.0000	ADAM9/ALCAM/BTN3A2/CD27/CD59/CD79B/ENG/FOLR1/GFRA1/IL1R1/IL1RL1/ITGA11/ITGAV/MFGE8/OSMR/SDC1/TGFBR2/THBD/THY1/TNFRSF11A/ULBP2	21
Health behaviors	CC	GO:0062023	collagen-containing extracellular matrix	22/157	428/19894	0.0514	6.5133	0.0000	0.0000	AGRN/AMBP/ANGPTL1/ANGPTL7/BCAN/CCDC80/CTSD/CTSH/EDIL3/GDF15/IGFBPL1/LAMA4/LGALS3BP/MFGE8/RARRES2/SERPING1/SFRP1/SMOC2/SPON1/THBS2/TIMP1/TNR	22
Health behaviors	CC	GO:0005775	vacuolar lumen	8/157	176/19894	0.0455	5.7597	0.0169	0.0015	ADA2/AGRN/BCAN/CTSD/HYAL1/MPO/SDC1/TPP1	8
Health behaviors	CC	GO:0034774	secretory granule lumen	12/157	322/19894	0.0373	4.7222	0.0021	0.0004	ADA2/CANT1/CHI3L1/CTSD/CTSH/FAM3C/LGALS3BP/MPO/QPCT/RARRES2/SERPING1/TIMP1	12

Supplementary Table 3 GO Enrichment Analysis Results for the Exposure Pattern Proteomic Signature, assessed using a one-sided Fisher's exact test with Bonferroni correction for multiple testing

Expposure pattern	ONTOLOGY	ID	Description	GeneRatio	BgRatio	RichFactor	FoldEnrichment	p.adjust	qvalue	geneID	Count
Health behaviors	CC	GO:0060205	cytoplasmic vesicle lumen	12/157	325/19894	0.0369	4.6786	0.0023	0.0004	ADA2/CANT1/CHI3L1/CTSD/CTSH/FAM3C/LGALS3BP/MPO/QPCT/RARRES2/SERPING1/TIMP1	12
Health behaviors	CC	GO:0031983	vesicle lumen	12/157	326/19894	0.0368	4.6643	0.0024	0.0004	ADA2/CANT1/CHI3L1/CTSD/CTSH/FAM3C/LGALS3BP/MPO/QPCT/RARRES2/SERPING1/TIMP1	12
Health behaviors	CC	GO:0005788	endoplasmic reticulum lumen	11/157	313/19894	0.0351	4.4532	0.0085	0.0009	ADAMTS13/FGF23/FSTL1/HYOU1/IGFBP1/IGFBP4/MFGE8/SERPING1/SPON1/STC2/TIMP1	11
Health behaviors	CC	GO:0005925	focal adhesion	13/157	421/19894	0.0309	3.9128	0.0065	0.0009	ADAM9/ALCAM/CD59/ENAH/ENG/EPHA2/HYOU1/IL1RL1/ITGA11/ITGAV/MME/THY1/TNFSF13B	13
Health behaviors	CC	GO:0030055	cell-substrate junction	13/157	431/19894	0.0302	3.8220	0.0083	0.0009	ADAM9/ALCAM/CD59/ENAH/ENG/EPHA2/HYOU1/IL1RL1/ITGA11/ITGAV/MME/THY1/TNFSF13B	13
Health behaviors	MF	GO:0031994	insulin-like growth factor I binding	4/154	13/18522	0.3077	37.0070	0.0009	0.0001	IGFBP1/IGFBP2/IGFBP4/ITGAV	4
Health behaviors	MF	GO:0048185	activin binding	3/154	11/18522	0.2727	32.8017	0.0259	0.0009	ACVRL1/ENG/TGFBR2	3
Health behaviors	MF	GO:0005520	insulin-like growth factor binding	5/154	19/18522	0.2632	31.6507	0.0001	0.0000	IGFBP1/IGFBP2/IGFBP4/IGFBPL1/ITGAV	5
Health behaviors	MF	GO:0050135	NADP+ nucleosidase activity	4/154	16/18522	0.2500	30.0682	0.0023	0.0001	CD38/IL18R1/IL1R1/IL1RL1	4
Health behaviors	MF	GO:0061809	NAD+ nucleotidase, cyclic ADP-ribose generating	4/154	16/18522	0.2500	30.0682	0.0023	0.0001	CD38/IL18R1/IL1R1/IL1RL1	4
Health behaviors	MF	GO:0005024	transforming growth factor beta receptor activity	3/154	13/18522	0.2308	27.7552	0.0444	0.0013	ACVRL1/ENG/TGFBR2	3
Health behaviors	MF	GO:0050431	transforming growth factor beta binding	4/154	24/18522	0.1667	20.0455	0.0126	0.0005	ACVRL1/ENG/ITGAV/TGFBR2	4
Health behaviors	MF	GO:0051861	glycolipid binding	4/154	29/18522	0.1379	16.5893	0.0272	0.0009	CEACAM5/THY1/TPP1/TREM2	4
Health behaviors	MF	GO:0008009	chemokine activity	6/154	49/18522	0.1224	14.7273	0.0009	0.0001	CCL11/CCL16/CCL20/CCL3/CX3CL1/CXCL13	6
Health behaviors	MF	GO:0048020	CCR chemokine receptor binding	6/154	50/18522	0.1200	14.4327	0.0010	0.0001	CCL11/CCL16/CCL20/CCL3/CX3CL1/CXCL13	6

Supplementary Table 3 GO Enrichment Analysis Results for the Exposure Pattern Proteomic Signature, assessed using a one-sided Fisher's exact test with Bonferroni correction for multiple testing

Expposure pattern	ONTOLOGY	ID	Description	GeneRatio	BgRatio	RichFactor	FoldEnrichment	p.adjust	qvalue	geneID	Count
Health behaviors	MF	GO:0019838	growth factor binding	13/154	135/18522	0.0963	11.5818	0.0000	0.0000	ACVRL1/CXCL13/ENG/EPHA2/FGFBP1/IGFBP1/IGFBP2/IGFBP4/IGFBPL1/IL1R1/ITGAV/OSMR/TGFBR2	13
Health behaviors	MF	GO:0042379	chemokine receptor binding	6/154	74/18522	0.0811	9.7518	0.0102	0.0004	CCL11/CCL16/CCL20/CCL3/CX3CL1/CXCL13	6
Health behaviors	MF	GO:0005539	glycosaminoglycan binding	17/154	239/18522	0.0711	8.5550	0.0000	0.0000	ADA2/AGRN/BCAN/CCDC80/CXCL13/ENG/FGFBP1/FSTL1/MPO/PTN/REG1A/REG4/SFRP1/SMOC2/TGFBR2/THBS2/TREM2	17
Health behaviors	MF	GO:0019955	cytokine binding	10/154	145/18522	0.0690	8.2947	0.0001	0.0000	ACVRL1/ENG/IL18R1/IL1R1/IL1RL1/ITGAV/NBL1/OSMR/TGFBR2/TNFRSF11A	10
Health behaviors	MF	GO:0005178	integrin binding	10/154	157/18522	0.0637	7.6607	0.0002	0.0000	ADAM9/ADAMTS13/CX3CL1/EDIL3/GFRA1/ITGA11/ITGAV/MFGE8/PTN/THY1	10
Health behaviors	MF	GO:0008201	heparin binding	11/154	174/18522	0.0632	7.6034	0.0001	0.0000	ADA2/CCDC80/CXCL13/FGFBP1/FSTL1/MPO/PTN/REG4/SFRP1/SMOC2/THBS2	11
Health behaviors	MF	GO:0004896	cytokine receptor activity	6/154	96/18522	0.0625	7.5170	0.0440	0.0013	GFRA1/IL10RB/IL18R1/IL1R1/IL1RL1/OSMR	6
Health behaviors	MF	GO:0140375	immune receptor activity	9/154	151/18522	0.0596	7.1686	0.0014	0.0001	CTSH/GFRA1/IL10RB/IL18R1/IL1R1/IL1RL1/LILRA5/LILRB4/OSMR	9
Health behaviors	MF	GO:0005125	cytokine activity	13/154	238/18522	0.0546	6.5695	0.0000	0.0000	CCL11/CCL16/CCL20/CCL3/CX3CL1/CXCL13/FAM3B/FAM3C/GDF15/IL17C/TAFA5/TIMP1/TNFSF13	13
Health behaviors	MF	GO:0030246	carbohydrate binding	13/154	279/18522	0.0466	5.6041	0.0002	0.0000	AMBP/BCAN/CD93/CHI3L1/ENG/FAM3B/FAM3C/GALNT10/PTN/REG1A/REG4/SIGLEC1/SIGLEC7	13
Health behaviors	MF	GO:0031406	carboxylic acid binding	9/154	196/18522	0.0459	5.5227	0.0115	0.0005	AGRN/AMBP/DDAH1/FABP3/FABP4/FOLR1/PTGDS/RBP2/SIGLEC7	9

Supplementary Table 3 GO Enrichment Analysis Results for the Exposure Pattern Proteomic Signature, assessed using a one-sided Fisher's exact test with Bonferroni correction for multiple testing

Expposure pattern	ONTOLOGY	ID	Description	GeneRatio	BgRatio	RichFactor	FoldEnrichment	p.adjust	qvalue	geneID	Count
Health behaviors	MF	GO:1901681	sulfur compound binding	12/154	267/18522	0.0449	5.4055	0.0007	0.0001	ADA2/AGRN/CCDC80/CXCL13/FGFBP1/FSTL1/MPO/PTN/REG4/SFRP1/SMOC2/THBS2	12
Health behaviors	MF	GO:0043177	organic acid binding	9/154	208/18522	0.0433	5.2041	0.0182	0.0007	AGRN/AMBP/DDAH1/FABP3/FABP4/FOLR1/PTGDS/RBP2/SIGLEC7	9
Health behaviors	MF	GO:0001664	G protein-coupled receptor binding	12/154	291/18522	0.0412	4.9597	0.0017	0.0001	ADA2/ADM/CCL11/CCL16/CCL20/CCL3/CX3CL1/CXCL13/GAL/MLN/SFRP1/TAFA5	12
Health behaviors	MF	GO:0005126	cytokine receptor binding	10/154	273/18522	0.0366	4.4056	0.0287	0.0009	CCL11/CCL16/CCL20/CCL3/CX3CL1/CXCL13/ENG/OSMR/TGFBR2/TNFSF13B	10
Health behaviors	MF	GO:0004175	endopeptidase activity	13/154	388/18522	0.0335	4.0298	0.0065	0.0003	ADAM9/ADAMTS13/C2/CTSD/CTSH/KLK10/KLK8/MME/MMP7/PLAT/REN/SFRP1/TPP1	13
Social deprivation	BP	GO:0048245	eosinophil chemotaxis	7/163	27/18888	0.2593	30.0423	0.0000	0.0000	CCL11/CCL15/CCL16/CCL22/CCL25/CCL3/CX3CL1	7
Social deprivation	BP	GO:0072677	eosinophil migration	8/163	31/18888	0.2581	29.9038	0.0000	0.0000	ADAM8/CCL11/CCL15/CCL16/CCL22/CCL25/CCL3/CX3CL1	8
Social deprivation	BP	GO:0035902	response to immobilization stress	5/163	26/18888	0.1923	22.2841	0.0074	0.0001	GAL/LRP11/REN/SOD2/TFF1	5
Social deprivation	BP	GO:0048247	lymphocyte chemotaxis	12/163	64/18888	0.1875	21.7270	0.0000	0.0000	ADAM8/CCL11/CCL15/CCL16/CCL20/CCL22/CCL25/CCL3/CX3CL1/CXCL10/CXCL13/CXCL16	12
Social deprivation	BP	GO:0002548	monocyte chemotaxis	13/163	70/18888	0.1857	21.5201	0.0000	0.0000	CCL11/CCL15/CCL16/CCL20/CCL22/CCL25/CCL3/CX3CL1/CXCL10/CXCL17/IL6/SLAMF8/TNFRSF11A	13
Social deprivation	BP	GO:0002675	positive regulation of acute inflammatory response	5/163	28/18888	0.1786	20.6924	0.0109	0.0001	ADAM8/IL6/OSMR/TNF/TNFRSF11A	5
Social deprivation	BP	GO:0043171	peptide catabolic process	5/163	33/18888	0.1515	17.5572	0.0254	0.0003	ADAMTS13/CTSH/GGT1/MME/TPP1	5
Social deprivation	BP	GO:0060055	angiogenesis involved in wound healing	5/163	33/18888	0.1515	17.5572	0.0254	0.0003	B4GALT1/CX3CL1/SMOC2/TAFA5/TNF	5

Supplementary Table 3 GO Enrichment Analysis Results for the Exposure Pattern Proteomic Signature, assessed using a one-sided Fisher's exact test with Bonferroni correction for multiple testing

Expposure pattern	ONTOLOGY	ID	Description	GeneRatio	BgRatio	RichFactor	FoldEnrichment	p.adjust	qvalue	geneID	Count
Social deprivation	BP	GO:0071347	cellular response to interleukin-1	14/163	110/18888	0.1273	14.7480	0.0000	0.0000	CCL11/CCL15/CCL16/CCL20/CCL22/CCL25/CCL3/CX3CL1/CXCL8/HYAL1/IL1R1/IL6/INHBB/SFRP1	14
Social deprivation	BP	GO:0070555	response to interleukin-1	17/163	138/18888	0.1232	14.2747	0.0000	0.0000	CCL11/CCL15/CCL16/CCL20/CCL22/CCL25/CCL3/CD38/CX3CL1/CXCL8/HYAL1/IL1R1/IL6/INHBB/SFRP1/SMPD1/TNFRSF11A	17
Social deprivation	BP	GO:0070098	chemokine-mediated signaling pathway	11/163	93/18888	0.1183	13.7059	0.0000	0.0000	CCL11/CCL15/CCL16/CCL20/CCL22/CCL25/CCL3/CX3CL1/CXCL10/CXCL13/CXCL8	11
Social deprivation	BP	GO:1990868	response to chemokine	11/163	101/18888	0.1089	12.6203	0.0000	0.0000	CCL11/CCL15/CCL16/CCL20/CCL22/CCL25/CCL3/CX3CL1/CXCL10/CXCL13/CXCL8	11
Social deprivation	BP	GO:1990869	cellular response to chemokine	11/163	101/18888	0.1089	12.6203	0.0000	0.0000	CCL11/CCL15/CCL16/CCL20/CCL22/CCL25/CCL3/CX3CL1/CXCL10/CXCL13/CXCL8	11
Social deprivation	BP	GO:1990266	neutrophil migration	14/163	129/18888	0.1085	12.5758	0.0000	0.0000	ADAM8/CCL11/CCL15/CCL16/CCL20/CCL22/CCL25/CCL3/CX3CL1/CXCL10/CXCL13/CXCL8/IL1R1/SLAMF8	14
Social deprivation	BP	GO:0097530	granulocyte migration	16/163	154/18888	0.1039	12.0392	0.0000	0.0000	ADAM8/CCL11/CCL15/CCL16/CCL20/CCL22/CCL25/CCL3/CX3CL1/CXCL10/CXCL13/CXCL17/CXCL8/IL1R1/RARRES2/SLAMF8	16
Social deprivation	BP	GO:0030593	neutrophil chemotaxis	11/163	107/18888	0.1028	11.9126	0.0000	0.0000	CCL11/CCL15/CCL16/CCL20/CCL22/CCL25/CCL3/CX3CL1/CXCL10/CXCL13/CXCL8	11
Social deprivation	BP	GO:0071621	granulocyte chemotaxis	13/163	128/18888	0.1016	11.7688	0.0000	0.0000	CCL11/CCL15/CCL16/CCL20/CCL22/CCL25/CCL3/CX3CL1/CXCL10/CXCL13/CXCL17/CXCL8/RARRES2	13

Supplementary Table 3 GO Enrichment Analysis Results for the Exposure Pattern Proteomic Signature, assessed using a one-sided Fisher's exact test with Bonferroni correction for multiple testing

Expposure pattern	ONTOLOGY	ID	Description	GeneRatio	BgRatio	RichFactor	FoldEnrichment	p.adjust	qvalue	geneID	Count
Social deprivation	BP	GO:0071346	cellular response to type II interferon	12/163	120/18888	0.1000	11.5877	0.0000	0.0000	ADAMTS13/CCL11/CCL15/CCL16/CCL20/CCL22/CCL25/CCL3/CX3CL1/IFNGR1/MRC1/TNF	12
Social deprivation	BP	GO:0050918	positive chemotaxis	7/163	70/18888	0.1000	11.5877	0.0069	0.0001	CCL15/CCL16/CCL3/CX3CL1/CXCL10/CXCL8/PGF	7
Social deprivation	BP	GO:1905517	macrophage migration	6/163	60/18888	0.1000	11.5877	0.0371	0.0004	B4GALT1/CCL3/CX3CL1/CXCL17/RARRES2/SLAMF8	6
Social deprivation	BP	GO:0034341	response to type II interferon	14/163	142/18888	0.0986	11.4245	0.0000	0.0000	ADAMTS13/BST2/CCL11/CCL15/CCL16/CCL20/CCL22/CCL25/CCL3/CX3CL1/CXCL16/IFNGR1/MRC1/TNF	14
Social deprivation	BP	GO:0071677	positive regulation of mononuclear cell migration	7/163	71/18888	0.0986	11.4245	0.0076	0.0001	ADAM8/CCL20/CCL3/CXCL10/CXCL13/CXCL17/TNF	7
Social deprivation	BP	GO:0033619	membrane protein proteolysis	6/163	62/18888	0.0968	11.2139	0.0450	0.0004	ADAM8/ADAM9/CTSH/MMP7/TIMP1/TNF	6
Social deprivation	BP	GO:0072676	lymphocyte migration	12/163	125/18888	0.0960	11.1242	0.0000	0.0000	ADAM8/CCL11/CCL15/CCL16/CCL20/CCL22/CCL25/CCL3/CX3CL1/CXCL10/CXCL13/CXCL16	12
Social deprivation	BP	GO:0002687	positive regulation of leukocyte migration	14/163	149/18888	0.0940	10.8878	0.0000	0.0000	ADAM8/CCL20/CCL3/CX3CL1/CXCL10/CXCL13/CXCL17/CXCL8/IL1R1/IL6/PGF/RARRES2/THY1/TNF	14
Social deprivation	BP	GO:0046849	bone remodeling	8/163	90/18888	0.0889	10.3002	0.0032	0.0000	ACP5/ADAM8/CD38/EPHA2/IL6/SFRP1/TNFRSF11A/TPP1	8
Social deprivation	BP	GO:0071674	mononuclear cell migration	18/163	206/18888	0.0874	10.1252	0.0000	0.0000	ADAM8/CCL11/CCL15/CCL16/CCL20/CCL22/CCL25/CCL3/CX3CL1/CXCL10/CXCL13/CXCL16/CXCL17/IL6/RARRES2/SLAMF8/TNF/TNFRSF11A	18
Social deprivation	BP	GO:0002690	positive regulation of leukocyte chemotaxis	8/163	93/18888	0.0860	9.9679	0.0041	0.0001	CCL3/CXCL10/CXCL13/CXCL17/CXCL8/IL6/PGF/RARRES2	8

Supplementary Table 3 GO Enrichment Analysis Results for the Exposure Pattern Proteomic Signature, assessed using a one-sided Fisher's exact test with Bonferroni correction for multiple testing

Expposure pattern	ONTOLOGY	ID	Description	GeneRatio	BgRatio	RichFactor	FoldEnrichment	p.adjust	qvalue	geneID	Count
Social deprivation	BP	GO:0097529	myeloid leukocyte migration	20/163	239/18888	0.0837	9.6968	0.0000	0.0000	ADAM8/B4GALT1/CCL11/CCL15/CCL16/CCL20/CCL22/CCL25/CCL3/CX3CL1/CXCL10/CXCL13/CXCL17/CXCL8/IL1R1/IL6/PGF/RARRES2/SLAMF8/TNFRSF11A	20
Social deprivation	BP	GO:0030595	leukocyte chemotaxis	19/163	237/18888	0.0802	9.2897	0.0000	0.0000	ADAM8/CCL11/CCL15/CCL16/CCL20/CCL22/CCL25/CCL3/CX3CL1/CXCL10/CXCL13/CXCL16/CXCL17/CXCL8/IL6/PGF/RARRES2/SLAMF8/TNFRSF11A	19
Social deprivation	BP	GO:0044344	cellular response to fibroblast growth factor stimulus	9/163	116/18888	0.0776	8.9905	0.0021	0.0000	CXCL13/CXCL8/FGF21/FGF23/FGFBP1/FGFR2/HYAL1/SFRP1/SMOC2	9
Social deprivation	BP	GO:0033273	response to vitamin	7/163	91/18888	0.0769	8.9136	0.0400	0.0004	CD40/CXCL10/FGF23/FOLR1/SFRP1/SOD2/STC1	7
Social deprivation	BP	GO:0002688	regulation of leukocyte chemotaxis	9/163	123/18888	0.0732	8.4788	0.0035	0.0001	CCL3/CXCL10/CXCL13/CXCL17/CXCL8/IL6/PGF/RARRES2/SLAM	9
Social deprivation	BP	GO:0071675	regulation of mononuclear cell migration	9/163	123/18888	0.0732	8.4788	0.0035	0.0001	ADAM8/CCL20/CCL3/CXCL10/CXCL13/CXCL17/RARRES2/SLAMF8/TNF	9
Social deprivation	BP	GO:0071774	response to fibroblast growth factor	9/163	124/18888	0.0726	8.4104	0.0037	0.0001	CXCL13/CXCL8/FGF21/FGF23/FGFBP1/FGFR2/HYAL1/SFRP1/SMOC2	9
Social deprivation	BP	GO:0034612	response to tumor necrosis factor	19/163	263/18888	0.0722	8.3714	0.0000	0.0000	ADAM9/ADAMTS13/CCL11/CCL15/CCL16/CCL20/CCL22/CCL25/CCL3/CX3CL1/CXCL16/CXCL8/HYAL1/KRT18/SFRP1/SMPD1/TNF/TNFRSF11A/TNFSF13B	19
Social deprivation	BP	GO:0002685	regulation of leukocyte migration	16/163	230/18888	0.0696	8.0610	0.0000	0.0000	ADAM8/CCL20/CCL25/CCL3/CX3CL1/CXCL10/CXCL13/CXCL17/CXCL8/IL1R1/IL6/PGF/RARRES2/SLAMF8/THY1/TNF	16

Supplementary Table 3 GO Enrichment Analysis Results for the Exposure Pattern Proteomic Signature, assessed using a one-sided Fisher's exact test with Bonferroni correction for multiple testing

Expposure pattern	ONTOLOGY	ID	Description	GeneRatio	BgRatio	RichFactor	FoldEnrichment	p.adjust	qvalue	geneID	Count
Social deprivation	BP	GO:0071356	cellular response to tumor necrosis factor	16/163	241/18888	0.0664	7.6931	0.0000	0.0000	ADAMTS13/CCL11/CCL15/CCL16/CCL20/CCL22/CCL25/CCL3/CX3CL1/CXCL8/HYAL1/KRT18/SFRP1/TNF/TNFRSF11A/TNFSF13B	16
Social deprivation	BP	GO:0060326	cell chemotaxis	21/163	322/18888	0.0652	7.5572	0.0000	0.0000	ADAM8/CCL11/CCL15/CCL16/CCL20/CCL22/CCL25/CCL3/CX3CL1/CXCL10/CXCL13/CXCL16/CXCL17/CXCL8/EPHA2/IL6/PGF/RARRES2/SLAMF8/SMOC2/TNFRSF11	21
Social deprivation	BP	GO:0050921	positive regulation of chemotaxis	9/163	142/18888	0.0634	7.3443	0.0116	0.0001	CCL3/CXCL10/CXCL13/CXCL17/CXCL8/IL6/PGF/RARRES2/SMOC	9
Social deprivation	BP	GO:0043547	positive regulation of GTPase activity	14/163	230/18888	0.0609	7.0534	0.0000	0.0000	AGRN/CCL11/CCL15/CCL16/CCL20/CCL22/CCL25/CCL3/CD40/CX3CL1/CXCL13/EPHA2/SFRP1/THY1	14
Social deprivation	BP	GO:0070374	positive regulation of ERK1 and ERK2 cascade	13/163	215/18888	0.0605	7.0065	0.0001	0.0000	CCL11/CCL15/CCL16/CCL20/CCL22/CCL25/CCL3/CX3CL1/CXCL17/FGF21/FGF23/FGFR2/TNF	13
Social deprivation	BP	GO:0045766	positive regulation of angiogenesis	11/163	187/18888	0.0588	6.8163	0.0020	0.0000	ACVRL1/CCL11/CD40/CTSH/CXCL8/DDAH1/ENG/HSPB6/HYAL1/PGF/SMOC2	11
Social deprivation	BP	GO:0050729	positive regulation of inflammatory response	9/163	153/18888	0.0588	6.8163	0.0213	0.0002	ADAM8/CCL3/CX3CL1/IL1RL1/IL6/LILRA5/OSMR/TNF/TNFRSF11A	9
Social deprivation	BP	GO:0048771	tissue remodeling	11/163	188/18888	0.0585	6.7801	0.0021	0.0000	ACP5/ACVRL1/ADAM8/CD38/EPHA2/IL6/NPPC/SFRP1/TIMP1/TNFRSF11A/TPP1	11
Social deprivation	BP	GO:0050900	leukocyte migration	23/163	396/18888	0.0581	6.7302	0.0000	0.0000	ADAM8/B4GALT1/CCL11/CCL15/CCL16/CCL20/CCL22/CCL25/CCL3/CX3CL1/CXCL10/CXCL13/CXCL16/CXCL17/CXCL8/IL1R1/IL6/PGF/RARRES2/SLAMF8/THY1/TNF/TNFRSF11A	23

Supplementary Table 3 GO Enrichment Analysis Results for the Exposure Pattern Proteomic Signature, assessed using a one-sided Fisher's exact test with Bonferroni correction for multiple testing

Expposure pattern	ONTOLOGY	ID	Description	GeneRatio	BgRatio	RichFactor	FoldEnrichment	p.adjust	qvalue	geneID	Count
Social deprivation	BP	GO:1904018	positive regulation of vasculature development	11/163	190/18888	0.0579	6.7087	0.0023	0.0000	ACVRL1/CCL11/CD40/CTSH/CXCL8/DDAH1/ENG/HSPB6/HYAL1/PGF/SMOC2	11
Social deprivation	BP	GO:0007160	cell-matrix adhesion	13/163	241/18888	0.0539	6.2506	0.0005	0.0000	ACVRL1/ADAM9/ADAMTS13/CCL25/CEACAM6/CX3CL1/ITGA11/ITGAV/ITGBL1/SFRP1/SIGLEC1/SORBS1/THY1	13
Social deprivation	BP	GO:0071222	cellular response to lipopolysaccharide	12/163	225/18888	0.0533	6.1801	0.0018	0.0000	ADAM9/ADAMTS13/CD274/CD40/CX3CL1/CXCL10/CXCL13/CXCL8/IL6/MRC1/SPON2/TNF	12
Social deprivation	BP	GO:1901342	regulation of vasculature development	19/163	362/18888	0.0525	6.0820	0.0000	0.0000	ACVRL1/ANGPTL7/CCL11/CD40/CTSH/CXCL10/CXCL13/CXCL8/DDAH1/ENG/EPHA2/HSPB6/HYAL1/IL6/PGF/SFRP1/SMOC2/TAFA5/TNF	19
Social deprivation	BP	GO:0050920	regulation of chemotaxis	12/163	229/18888	0.0524	6.0722	0.0021	0.0000	CCL3/CXCL10/CXCL13/CXCL17/CXCL8/IL6/PGF/RARRES2/SEMA3F/SLAMF8/SMOC2/ST6GAL1	12
Social deprivation	BP	GO:0006935	chemotaxis	24/163	466/18888	0.0515	5.9679	0.0000	0.0000	ADAM8/CCL11/CCL15/CCL16/CCL20/CCL22/CCL25/CCL3/CX3CL1/CXCL10/CXCL13/CXCL16/CXCL17/CXCL8/EPHA2/IL6/ITGAV/PGF/RARRES2/SEMA3F/SLAMF8/SMOC2/ST6GAL1/TNFRSF11A	24
Social deprivation	BP	GO:0042330	taxis	24/163	468/18888	0.0513	5.9424	0.0000	0.0000	ADAM8/CCL11/CCL15/CCL16/CCL20/CCL22/CCL25/CCL3/CX3CL1/CXCL10/CXCL13/CXCL16/CXCL17/CXCL8/EPHA2/IL6/ITGAV/PGF/RARRES2/SEMA3F/SLAMF8/SMOC2/ST6GAL1/TNFRSF11A	24

Supplementary Table 3 GO Enrichment Analysis Results for the Exposure Pattern Proteomic Signature, assessed using a one-sided Fisher's exact test with Bonferroni correction for multiple testing

Expposure pattern	ONTOLOGY	ID	Description	GeneRatio	BgRatio	RichFactor	FoldEnrichment	p.adjust	qvalue	geneID	Count
Social deprivation	BP	GO:0045765	regulation of angiogenesis	18/163	353/18888	0.0510	5.9088	0.0000	0.0000	ACVRL1/CCL11/CD40/CTSH/CXCL10/CXCL13/CXCL8/DDAH1/ENG/EPHA2/HSPB6/HYAL1/IL6/PGF/SFRP1/SMOC2/TAFA5/TNF	18
Social deprivation	BP	GO:0071219	cellular response to molecule of bacterial origin	12/163	238/18888	0.0504	5.8426	0.0032	0.0000	ADAM9/ADAMTS13/CD274/CD40/CX3CL1/CXCL10/CXCL13/CXCL8/IL6/MRC1/SPON2/TNF	12
Social deprivation	BP	GO:0071216	cellular response to biotic stimulus	13/163	265/18888	0.0491	5.6845	0.0015	0.0000	ADAM9/ADAMTS13/CD274/CD40/CX3CL1/CXCL10/CXCL13/CXCL8/IGFBPL1/IL6/MRC1/SPON2/TNF	13
Social deprivation	BP	GO:0032496	response to lipopolysaccharide	17/163	348/18888	0.0489	5.6607	0.0000	0.0000	ACP5/ADAM9/ADAMTS13/CD274/CD40/CX3CL1/CXCL10/CXCL13/CXCL8/FGFR2/IL6/MRC1/REN/SOD2/SPON2/TNF/TNFRSF11A	17
Social deprivation	BP	GO:0043410	positive regulation of MAPK cascade	23/163	475/18888	0.0484	5.6109	0.0000	0.0000	ADAM8/ADAM9/CCL11/CCL15/CCL16/CCL20/CCL22/CCL25/CCL3/CD40/CDH2/CX3CL1/CXCL17/FGF21/FGF23/FGFR2/GDF15/GPR37/IGFBP4/IL6/LILRA5/TNF/TNFRSF11A	23
Social deprivation	BP	GO:0070371	ERK1 and ERK2 cascade	16/163	332/18888	0.0482	5.5844	0.0001	0.0000	CCL11/CCL15/CCL16/CCL20/CCL22/CCL25/CCL3/CTSH/CX3CL1/CXCL17/EPHA2/FGF21/FGF23/FGFR2/ITGAV/TNF	16
Social deprivation	BP	GO:0043087	regulation of GTPase activity	14/163	298/18888	0.0470	5.4439	0.0009	0.0000	AGRN/CCL11/CCL15/CCL16/CCL20/CCL22/CCL25/CCL3/CD40/CX3CL1/CXCL13/EPHA2/SFRP1/THY1	14
Social deprivation	BP	GO:0002237	response to molecule of bacterial origin	17/163	369/18888	0.0461	5.3385	0.0001	0.0000	ACP5/ADAM9/ADAMTS13/CD274/CD40/CX3CL1/CXCL10/CXCL13/CXCL8/FGFR2/IL6/MRC1/REN/SOD2/SPON2/TNF/TNFRSF11A	17

Supplementary Table 3 GO Enrichment Analysis Results for the Exposure Pattern Proteomic Signature, assessed using a one-sided Fisher's exact test with Bonferroni correction for multiple testing

Expposure pattern	ONTOLOGY	ID	Description	GeneRatio	BgRatio	RichFactor	FoldEnrichment	p.adjust	qvalue	geneID	Count
Social deprivation	BP	GO:0070372	regulation of ERK1 and ERK2 cascade	14/163	308/18888	0.0455	5.2672	0.0014	0.0000	CCL11/CCL15/CCL16/CCL20/CCL22/CCL25/CCL3/CX3CL1/CXCL17/EPHA2/FGF21/FGF23/FGFR2/TNF	14
Social deprivation	BP	GO:0031589	cell-substrate adhesion	16/163	359/18888	0.0446	5.1644	0.0003	0.0000	ACVRL1/ADAM9/ADAMTS13/AGR2/CCL25/CEACAM6/CX3CL1/EDIL3/ITGA11/ITGAV/ITGBL1/SFRP1/SIGLEC1/SORBS1/ST6GAL1/THY1	16
Social deprivation	BP	GO:0006959	humoral immune response	11/163	258/18888	0.0426	4.9405	0.0445	0.0004	ANG/CXCL10/CXCL13/CXCL8/IL6/PI3/RARRES2/SERPING1/SPON2/ST6GAL1/TNF	11
Social deprivation	BP	GO:0097305	response to alcohol	11/163	259/18888	0.0425	4.9214	0.0461	0.0004	ABCA2/CCL3/FGFR2/INHBB/NEFL/NPPC/SFRP1/SOD2/ST6GAL1/TNF/TNFRSF11A	11
Social deprivation	BP	GO:0001666	response to hypoxia	13/163	313/18888	0.0415	4.8128	0.0098	0.0001	ACVRL1/ADAM8/ANG/CD38/DDAH1/FGFR2/HYOU1/NPPC/PGF/SFRP1/SOD2/STC1/TNF	13
Social deprivation	BP	GO:0036293	response to decreased oxygen levels	13/163	327/18888	0.0398	4.6067	0.0157	0.0002	ACVRL1/ADAM8/ANG/CD38/DDAH1/FGFR2/HYOU1/NPPC/PGF/SFRP1/SOD2/STC1/TNF	13
Social deprivation	BP	GO:0051345	positive regulation of hydrolase activity	19/163	482/18888	0.0394	4.5678	0.0001	0.0000	AGRN/ANG/CCL11/CCL15/CCL16/CCL20/CCL22/CCL25/CCL3/CD40/CTSD/CTSH/CX3CL1/CXCL13/EPHA2/FGFR2/SFRP1/THY1/TN	19
Social deprivation	BP	GO:0030198	extracellular matrix organization	13/163	332/18888	0.0392	4.5374	0.0185	0.0002	ADAM8/ADAMTS13/ADAMTS4/ADAMTSL2/ANGPTL7/B4GALT1/ENG/IL6/MMP7/SMOC2/TNF/TNR/VWA1	13
Social deprivation	BP	GO:0043062	extracellular structure organization	13/163	333/18888	0.0390	4.5237	0.0191	0.0002	ADAM8/ADAMTS13/ADAMTS4/ADAMTSL2/ANGPTL7/B4GALT1/ENG/IL6/MMP7/SMOC2/TNF/TNR/VWA1	13

Supplementary Table 3 GO Enrichment Analysis Results for the Exposure Pattern Proteomic Signature, assessed using a one-sided Fisher's exact test with Bonferroni correction for multiple testing

Expposure pattern	ONTOLOGY	ID	Description	GeneRatio	BgRatio	RichFactor	FoldEnrichment	p.adjust	qvalue	geneID	Count
Social deprivation	BP	GO:0045229	external encapsulating structure organization	13/163	334/18888	0.0389	4.5102	0.0198	0.0002	ADAM8/ADAMTS13/ADAMTS4/ADAMTSL2/ANGPTL7/B4GALT1/ENG/IL6/MMP7/SMOC2/TNF/TNR/VWA1	13
Social deprivation	BP	GO:0045785	positive regulation of cell adhesion	18/163	485/18888	0.0371	4.3006	0.0006	0.0000	ADAM8/ADAM9/AGR2/CCL25/CD274/CD5/CEACAM6/CX3CL1/CXCL13/EDIL3/HYAL1/IGFBP2/IL6/ITGAV/SFRP1/THY1/TNF/TNFSF13B	18
Social deprivation	BP	GO:0070482	response to oxygen levels	13/163	354/18888	0.0367	4.2554	0.0368	0.0004	ACVRL1/ADAM8/ANG/CD38/DDAH1/FGFR2/HYOU1/NPPC/PGF/SFRP1/SOD2/STC1/TNF	13
Social deprivation	BP	GO:0050727	regulation of inflammatory response	15/163	425/18888	0.0353	4.0898	0.0125	0.0002	ACP5/ADAM8/CCL3/CX3CL1/CXCL17/GGT1/IL1R1/IL1RL1/IL22/IL6/LILRA5/OSMR/SLAMF8/TNF/TNFRSF11A	15
Social deprivation	BP	GO:0040013	negative regulation of locomotion	15/163	431/18888	0.0348	4.0329	0.0148	0.0002	ACVRL1/BST2/CCL25/CX3CL1/CXCL13/ENG/SEMA3F/SFRP1/SLAMF8/ST6GAL1/STC1/TAFA5/THY1/TIMP1/TNF	15
Social deprivation	BP	GO:0031667	response to nutrient levels	16/163	490/18888	0.0327	3.7837	0.0160	0.0002	CD40/CXCL10/FGF21/FGF23/FOLR1/GAST/GDF15/IGFBP2/INHBB/LRP11/NPPC/SFRP1/SOD2/STC1/TNF/TNFRSF11A	16
Social deprivation	BP	GO:0001667	ameboidal-type cell migration	16/163	498/18888	0.0321	3.7230	0.0196	0.0002	ACVRL1/ADAM9/CD40/CDH2/CTSH/CXCL13/EPHA2/FGFBP1/FOLR1/FSTL1/HYAL1/SEMA3F/SMOC2/STC1/TIMP1/TNF	16
Social deprivation	CC	GO:0031089	platelet dense granule lumen	3/166	14/19894	0.2143	25.6807	0.0437	0.0033	FAM3C/LGALS3BP/RARRES2	3
Social deprivation	CC	GO:0043202	lysosomal lumen	7/166	98/19894	0.0714	8.5602	0.0041	0.0006	AGRN/BCAN/CTSD/HYAL1/IFI30/SMPD1/TPP1	7
Social deprivation	CC	GO:0005604	basement membrane	6/166	92/19894	0.0652	7.8159	0.0274	0.0028	AGRN/ANG/LAMA4/SMOC2/TIMP1/VWA1	6

Supplementary Table 3 GO Enrichment Analysis Results for the Exposure Pattern Proteomic Signature, assessed using a one-sided Fisher's exact test with Bonferroni correction for multiple testing

Expposure pattern	ONTOLOGY	ID	Description	GeneRatio	BgRatio	RichFactor	FoldEnrichment	p.adjust	qvalue	geneID	Count
Social deprivation	CC	GO:0009897	external side of plasma membrane	22/166	405/19894	0.0543	6.5100	0.0000	0.0000	ADAM9/ALCAM/B4GALT1/BTN3A2/CD274/CD40/CD5/CRLF1/CXCL10/ENG/FOLR1/GFRA1/IL1R1/IL1RL1/ITGA11/ITGAV/MFGE8/OSMR/THY1/TNF/TNFRSF11A/ULBP2	22
Social deprivation	CC	GO:0062023	collagen-containing extracellular matrix	23/166	428/19894	0.0537	6.4402	0.0000	0.0000	ADAMTS4/AGRN/AMBP/ANG/ANGPTL7/BCAN/CDH2/CTSD/CTSH/EDIL3/FGFR2/GDF15/IGFBPL1/LAMA4/LGALS3BP/MFGE8/RARRES2/SERPING1/SFRP1/SMOC2/TIMP1/TNR/VWA1	23
Social deprivation	CC	GO:0005775	vacuolar lumen	8/166	176/19894	0.0455	5.4474	0.0265	0.0028	ADA2/AGRN/BCAN/CTSD/HYAL1/IFI30/SMPD1/TPP1	8
Social deprivation	CC	GO:0005788	endoplasmic reticulum lumen	13/166	313/19894	0.0415	4.9775	0.0005	0.0001	ADAMTS13/CDH2/FGF23/FSTL1/HYOU1/IGFBP1/IGFBP4/IL6/MFGE8/MZB1/SERPING1/TIMP1/VWA1	13
Social deprivation	CC	GO:0005925	focal adhesion	15/166	421/19894	0.0356	4.2700	0.0006	0.0001	ADAM9/ALCAM/CDH2/ENAH/ENG/EPHA2/HYOU1/IL1RL1/ITGA11/ITGAV/ITGBL1/MME/SORBS1/THY1/TNFSF13B	15
Social deprivation	CC	GO:0045121	membrane raft	10/166	286/19894	0.0350	4.1903	0.0336	0.0029	BST2/CDH2/CTSD/MME/SORBS1/THY1/TNF/TNFRSF11A/TNR/TPP1	10
Social deprivation	CC	GO:0098857	membrane microdomain	10/166	287/19894	0.0348	4.1757	0.0346	0.0029	BST2/CDH2/CTSD/MME/SORBS1/THY1/TNF/TNFRSF11A/TNR/TPP1	10
Social deprivation	CC	GO:0030055	cell-substrate junction	15/166	431/19894	0.0348	4.1709	0.0008	0.0001	ADAM9/ALCAM/CDH2/ENAH/ENG/EPHA2/HYOU1/IL1RL1/ITGA11/ITGAV/ITGBL1/MME/SORBS1/THY1/TNFSF13B	15
Social deprivation	MF	GO:0031994	insulin-like growth factor I binding	4/164	13/18522	0.3077	34.7505	0.0012	0.0001	IGFBP1/IGFBP2/IGFBP4/ITGAV	4

Supplementary Table 3 GO Enrichment Analysis Results for the Exposure Pattern Proteomic Signature, assessed using a one-sided Fisher's exact test with Bonferroni correction for multiple testing

Expposure pattern	ONTOLOGY	ID	Description	GeneRatio	BgRatio	RichFactor	FoldEnrichment	p.adjust	qvalue	geneID	Count
Social deprivation	MF	GO:0005520	insulin-like growth factor binding	5/164	19/18522	0.2632	29.7208	0.0002	0.0000	IGFBP1/IGFBP2/IGFBP4/IGFBPL1/ITGAV	5
Social deprivation	MF	GO:0050135	NADP+ nucleosidase activity	4/164	16/18522	0.2500	28.2348	0.0029	0.0001	CD38/IL18R1/IL1R1/IL1RL1	4
Social deprivation	MF	GO:0061809	NAD+ nucleotidase, cyclic ADP-ribose generating	4/164	16/18522	0.2500	28.2348	0.0029	0.0001	CD38/IL18R1/IL1R1/IL1RL1	4
Social deprivation	MF	GO:0008009	chemokine activity	12/164	49/18522	0.2449	27.6585	0.0000	0.0000	CCL11/CCL15/CCL16/CCL20/CCL22/CCL25/CCL3/CX3CL1/CXCL10/CXCL13/CXCL16/CXCL8	12
Social deprivation	MF	GO:0045236	CXCR chemokine receptor binding	4/164	18/18522	0.2222	25.0976	0.0049	0.0002	CX3CL1/CXCL10/CXCL13/CXCL8	4
Social deprivation	MF	GO:0048020	CCR chemokine receptor binding	9/164	50/18522	0.1800	20.3290	0.0000	0.0000	CCL11/CCL15/CCL16/CCL20/CCL22/CCL25/CCL3/CX3CL1/CXCL13	9
Social deprivation	MF	GO:0017134	fibroblast growth factor binding	4/164	23/18522	0.1739	19.6416	0.0137	0.0004	CXCL13/FGFBP1/FGFR2/ITGAV	4
Social deprivation	MF	GO:0042379	chemokine receptor binding	12/164	74/18522	0.1622	18.3144	0.0000	0.0000	CCL11/CCL15/CCL16/CCL20/CCL22/CCL25/CCL3/CX3CL1/CXCL10/CXCL13/CXCL16/CXCL8	12
Social deprivation	MF	GO:0042056	chemoattractant activity	6/164	41/18522	0.1463	16.5277	0.0005	0.0000	CCL15/CCL16/CCL3/CX3CL1/CXCL10/PGF	6
Social deprivation	MF	GO:0005125	cytokine activity	25/164	238/18522	0.1050	11.8633	0.0000	0.0000	CCL11/CCL15/CCL16/CCL20/CCL22/CCL25/CCL3/CRLF1/CX3CL1/CXCL10/CXCL13/CXCL16/CXCL8/FAM3B/FAM3C/GDF15/IL17C/IL19/IL22/IL6/INHBB/TAFA5/TIMP1/TNF/TNFSF13B	25
Social deprivation	MF	GO:0019838	growth factor binding	13/164	135/18522	0.0963	10.8756	0.0000	0.0000	ACVRL1/CXCL13/ENG/EPHA2/FGFBP1/FGFR2/IGFBP1/IGFBP2/IGFBP4/IGFBPL1/IL1R1/ITGAV/OSMR	13
Social deprivation	MF	GO:0050840	extracellular matrix binding	6/164	64/18522	0.0938	10.5880	0.0064	0.0002	ADAM9/ADAMTSL2/AGRN/ITGAV/SMOC2/VWA1	6
Social deprivation	MF	GO:0004896	cytokine receptor activity	8/164	96/18522	0.0833	9.4116	0.0006	0.0000	CRLF1/GFRA1/IFNGR1/IL10RB/IL18R1/IL1R1/IL1RL1/OSMR	8

Supplementary Table 3 GO Enrichment Analysis Results for the Exposure Pattern Proteomic Signature, assessed using a one-sided Fisher's exact test with Bonferroni correction for multiple testing

Expposure pattern	ONTOLOGY	ID	Description	GeneRatio	BgRatio	RichFactor	FoldEnrichment	p.adjust	qvalue	geneID	Count
Social deprivation	MF	GO:0008201	heparin binding	13/164	174/18522	0.0747	8.4380	0.0000	0.0000	ADA2/ANG/CCL15/CCN5/CXCL10/CXCL13/CXCL8/FGFBP1/FGFR2/FSTL1/PGF/SFRP1/SMOC2	13
Social deprivation	MF	GO:0005126	cytokine receptor binding	20/164	273/18522	0.0733	8.2739	0.0000	0.0000	CCL11/CCL15/CCL16/CCL20/CCL22/CCL25/CCL3/CRLF1/CX3CL1/CXCL10/CXCL13/CXCL16/CXCL8/ENG/IL22/IL6/OSMR/PGF/TNF/TNFSF13B	20
Social deprivation	MF	GO:0140375	immune receptor activity	11/164	151/18522	0.0728	8.2273	0.0000	0.0000	CRLF1/CTSH/GFRA1/IFNGR1/IL10RB/IL18R1/IL1R1/IL1RL1/LILRA5/OSMR/PIGR	11
Social deprivation	MF	GO:0005178	integrin binding	11/164	157/18522	0.0701	7.9129	0.0000	0.0000	ADAM9/ADAMTS13/CCN5/CX3CL1/EDIL3/GFRA1/ITGA11/ITGAV/ITGBL1/MFGE8/THY1	11
Social deprivation	MF	GO:0019955	cytokine binding	10/164	145/18522	0.0690	7.7889	0.0002	0.0000	ACVRL1/CRLF1/ENG/IFNGR1/IL18R1/IL1R1/IL1RL1/ITGAV/OSMR/TNFRSF11A	10
Social deprivation	MF	GO:0005539	glycosaminoglycan binding	16/164	239/18522	0.0669	7.5608	0.0000	0.0000	ADA2/AGRN/ANG/BCAN/CCL15/CCN5/CXCL10/CXCL13/CXCL8/ENG/FGFBP1/FGFR2/FSTL1/PGF/SFRP1/SMOC2	16
Social deprivation	MF	GO:0001664	G protein-coupled receptor binding	17/164	291/18522	0.0584	6.5978	0.0000	0.0000	ADA2/CCL11/CCL15/CCL16/CCL20/CCL22/CCL25/CCL3/CX3CL1/CXCL10/CXCL13/CXCL16/CXCL8/GAL/MLN/SFRP1/TAFA5	17
Social deprivation	MF	GO:0008083	growth factor activity	9/164	162/18522	0.0556	6.2744	0.0042	0.0002	ADA2/FGF21/FGF23/GDF15/IL6/INHBB/PGF/TFF1/TIMP1	9
Social deprivation	MF	GO:0005179	hormone activity	7/164	126/18522	0.0556	6.2744	0.0389	0.0011	CCL25/GAL/GAST/INHBB/MLN/NPPC/STC1	7
Social deprivation	MF	GO:1901681	sulfur compound binding	14/164	267/18522	0.0524	5.9219	0.0000	0.0000	ADA2/AGRN/ANG/CCL15/CCN5/CXCL10/CXCL13/CXCL8/FGFBP1/FGFR2/FSTL1/PGF/SFRP1/SMOC2	14

Supplementary Table 3 GO Enrichment Analysis Results for the Exposure Pattern Proteomic Signature, assessed using a one-sided Fisher's exact test with Bonferroni correction for multiple testing

Expposure pattern	ONTOLOGY	ID	Description	GeneRatio	BgRatio	RichFactor	FoldEnrichment	p.adjust	qvalue	geneID	Count
Social deprivation	MF	GO:0031406	carboxylic acid binding	9/164	196/18522	0.0459	5.1860	0.0190	0.0006	AGRN/AMBP/APOC1/DDAH1/FOLR1/RBP2/RBP5/SIGLEC7/SIGLEC8	9
Social deprivation	MF	GO:0043177	organic acid binding	9/164	208/18522	0.0433	4.8868	0.0299	0.0009	AGRN/AMBP/APOC1/DDAH1/FOLR1/RBP2/RBP5/SIGLEC7/SIGLEC8	9
Social deprivation	MF	GO:0030246	carbohydrate binding	11/164	279/18522	0.0394	4.4528	0.0118	0.0004	AMBP/BCAN/CD93/ENG/FAM3B/FAM3C/GALNT10/MRC1/SIGLEC1/SIGLEC7/SIGLEC8	11

Supplementary Table 4 KEGG Enrichment Analysis Results for the Exposure Pattern Proteomic Signature, assessed using a one-sided Fisher's exact test with Bonferroni correction for multiple testing

Exposure pattern	category	subcategory	ID	Description	GeneRatio	BgRatio	RichFactor	FoldEnrichment	p.adjust	qvalue	geneID	Count
Social deprivation	Environmental Information Processing	Signaling molecules and interaction	hsa04060	Cytokine-cytokine receptor interaction	33/108	298/8859	0.1107	9.0836	0.0000	0.0000	94/6356/6359/6360/6364/6367/6370/6348/958/6376/3627/10563/58191/284340/3576/9518/3459/3588/27189/8809/29949/3554/9173/50616/3569/3625/9180/7124/8794/8792/51330/8771/10673	33
Social deprivation	Environmental Information Processing	Signaling molecules and interaction	hsa04061	Viral protein interaction with cytokine and cytokine receptor	17/108	100/8859	0.1700	13.9447	0.0000	0.0000	6356/6359/6360/6364/6367/6370/6348/6376/3627/10563/3576/3588/8809/29949/3569/7124/8794	17
Social deprivation	Organismal Systems	Immune system	hsa04062	Chemokine signaling pathway	12/108	193/8859	0.0622	5.1002	0.0002	0.0002	6356/6359/6360/6364/6367/6370/6348/6376/3627/10563/58191/3576	12
Social deprivation	Human Diseases	Immune disease	hsa05323	Rheumatoid arthritis	8/108	95/8859	0.0842	6.9076	0.0009	0.0007	54/6364/6348/3576/3569/7124/8792/10673	8
Social deprivation	Organismal Systems	Immune system	hsa04657	IL-17 signaling pathway	7/108	95/8859	0.0737	6.0442	0.0056	0.0047	6356/6364/3627/3576/27189/3569/7124	7
Social deprivation	Cellular Processes	Transport and catabolism	hsa04142	Lysosome	8/108	132/8859	0.0606	4.9714	0.0061	0.0052	20/54/1509/1512/3373/23659/6609/1200	8
Social deprivation	Organismal Systems	Immune system	hsa04672	Intestinal immune network for IgA production	5/108	50/8859	0.1000	8.2028	0.0089	0.0075	6370/958/3569/5284/10673	5
Social deprivation	Human Diseases	Immune disease	hsa05321	Inflammatory bowel disease	5/108	66/8859	0.0758	6.2142	0.0277	0.0235	3459/8809/50616/3569/7124	5
Social deprivation	Organismal Systems	Immune system	hsa04640	Hematopoietic cell lineage	6/108	100/8859	0.0600	4.9217	0.0277	0.0235	952/921/3554/3569/4311/7124	6

Supplementary Table 4 KEGG Enrichment Analysis Results for the Exposure Pattern Proteomic Signature, assessed using a one-sided Fisher's exact test with Bonferroni correction for multiple testing

Exposure pattern	category	subcategory	ID	Description	GeneRatio	BgRatio	RichFactor	FoldEnrichment	p.adjust	qvalue	geneID	Count
Social deprivation	Environmental Information Processing	Signal transduction	hsa04064	NF-kappa B signaling pathway	6/108	105/8859	0.0571	4.6873	0.0320	0.0271	958/3576/3554/7124/8792/10673	6
Social deprivation	Organismal Systems	Immune system	hsa04620	Toll-like receptor signaling pathway	6/108	109/8859	0.0550	4.5153	0.0352	0.0299	6348/958/3627/3576/3569/7124	6
Social deprivation	Environmental Information Processing	Signaling molecules and interaction	hsa04514	Cell adhesion molecules	7/108	158/8859	0.0443	3.6341	0.0430	0.0365	214/29126/958/1000/3685/6614/114798	7
Social deprivation	Human Diseases	Infectious disease: parasitic	hsa05144	Malaria	4/108	50/8859	0.0800	6.5622	0.0430	0.0365	958/3576/3569/7124	4
Social deprivation	Environmental Information Processing	Signal transduction	hsa04668	TNF signaling pathway	6/108	119/8859	0.0504	4.1359	0.0430	0.0365	6364/6376/3627/8809/3569/7124	6
Blue and green spaces	Environmental Information Processing	Signaling molecules and interaction	hsa04060	Cytokine-cytokine receptor interaction	26/96	298/8859	0.0872	8.0514	0.0000	0.0000	94/6356/6360/6367/6348/6376/10563/284340/3576/10913/9518/3459/3588/27189/8809/29949/3554/9173/3569/9180/8797/8794/8792/51330/8771/10673	26
Blue and green spaces	Environmental Information Processing	Signaling molecules and interaction	hsa04061	Viral protein interaction with cytokine and cytokine receptor	13/96	100/8859	0.1300	11.9966	0.0000	0.0000	6356/6360/6367/6348/6376/10563/3576/3588/8809/29949/3569/8797/8794	13
Blue and green spaces	Cellular Processes	Transport and catabolism	hsa04142	Lysosome	7/96	132/8859	0.0530	4.8937	0.0298	0.0249	20/1509/1512/1519/3373/6609/1200	7

Supplementary Table 4 KEGG Enrichment Analysis Results for the Exposure Pattern Proteomic Signature, assessed using a one-sided Fisher's exact test with Bonferroni correction for multiple testing

Exposure pattern	category	subcategory	ID	Description	GeneRatio	BgRatio	RichFactor	FoldEnrichment	p.adjust	qvalue	geneID	Count
Health behaviors	Environmental Information Processing	Signaling molecules and interaction	hsa04060	Cytokine-cytokine receptor interaction	23/98	298/8859	0.0772	6.9770	0.0000	0.0000	94/6356/6360/6364/6348/939/6376/10563/284340/60401/9518/3588/27189/8809/3554/9173/9180/7048/8794/8792/51330/8771/10673	23
Health behaviors	Environmental Information Processing	Signaling molecules and interaction	hsa04061	Viral protein interaction with cytokine and cytokine receptor	9/98	100/8859	0.0900	8.1358	0.0001	0.0001	6356/6360/6364/6348/6376/10563/3588/8809/8794	9
Health behaviors	Environmental Information Processing	Signaling molecules and interaction	hsa04512	ECM-receptor interaction	7/98	89/8859	0.0787	7.1100	0.0025	0.0023	375790/22801/3685/3910/6382/7058/7143	7
Health behaviors	Organismal Systems	Development and regeneration	hsa04380	Osteoclast differentiation	7/98	143/8859	0.0490	4.4251	0.0351	0.0327	54/3554/353514/11006/7048/8792/54209	7
Air and noise pollution	Environmental Information Processing	Signaling molecules and interaction	hsa04060	Cytokine-cytokine receptor interaction	20/66	298/8859	0.0671	9.0085	0.0000	0.0000	94/6356/6360/6348/6376/10563/284340/3576/9518/3588/27189/8809/3554/9173/3569/9180/8794/8792/51330/8771	20
Air and noise pollution	Environmental Information Processing	Signaling molecules and interaction	hsa04061	Viral protein interaction with cytokine and cytokine receptor	10/66	100/8859	0.1000	13.4227	0.0000	0.0000	6356/6360/6348/6376/10563/3576/3588/8809/3569/8794	10

Supplementary Table 5 Associations between Individual Exposures and Cardiometabolic Diseases using Cox models adjusted with categorized Age and BMI, with significance assessed by two-sided Wald tests and Bonferroni correction.

Outcome	Exposure	HR(95% CI)	Adjusted p-value
Cerebrovascular Disease	Blue space	1.01(1.00 ,1.03)	1
Cerebrovascular Disease	Current Smoke	1.74(1.65 ,1.83)	1.37E-102
Cerebrovascular Disease	Depression/anxiety	1.33(1.28 ,1.38)	2.98E-49
Cerebrovascular Disease	Diet	0.97(0.95 ,0.98)	0.001556461
Cerebrovascular Disease	Green space	0.95(0.94 ,0.97)	2.05E-07
Cerebrovascular Disease	IMD scores	1.13(1.12 ,1.15)	3.50E-59
Cerebrovascular Disease	Leisure PA	0.96(0.94 ,0.97)	4.02E-06
Cerebrovascular Disease	NO2	1.07(1.06 ,1.09)	5.08E-17
Cerebrovascular Disease	Noise	1.02(1.00 ,1.03)	0.834803333
Cerebrovascular Disease	NOx	1.06(1.05 ,1.08)	5.71E-14
Cerebrovascular Disease	PM10	1.04(1.03 ,1.06)	3.61E-06
Cerebrovascular Disease	PM2.5	1.06(1.04 ,1.08)	2.54E-12
Cerebrovascular Disease	Previous Smoke	1.16(1.12 ,1.20)	5.14E-15
Cerebrovascular Disease	Sedentory behavior	1.09(1.07 ,1.11)	6.00E-25
Cerebrovascular Disease	Sleep duration	0.88(0.85 ,0.91)	6.53E-12
Cerebrovascular Disease	Social isolation	1.07(1.05 ,1.09)	3.27E-15
Cerebrovascular Disease	Total PA	1.01(0.99 ,1.02)	1
Death	Blue space	1.00(0.99 ,1.01)	1
Death	Current Smoke	2.61(2.52 ,2.71)	0
Death	Depression/anxiety	1.37(1.33 ,1.41)	1.01E-97
Death	Diet	0.92(0.91 ,0.93)	3.29E-31
Death	Green space	0.97(0.95 ,0.98)	3.78E-06
Death	IMD scores	1.20(1.18 ,1.21)	4.58E-211
Death	Leisure PA	0.87(0.86 ,0.88)	1.30E-75
Death	NO2	1.08(1.07 ,1.10)	4.79E-34
Death	Noise	1.03(1.02 ,1.04)	8.63E-05
Death	NOx	1.08(1.07 ,1.10)	2.89E-40
Death	PM10	1.03(1.02 ,1.05)	1.53E-06
Death	PM2.5	1.10(1.08 ,1.11)	3.96E-46
Death	Previous Smoke	1.41(1.37 ,1.45)	1.21E-120
Death	Sedentory behavior	1.14(1.13 ,1.16)	8.21E-106
Death	Sleep duration	0.83(0.81 ,0.86)	4.13E-39
Death	Social isolation	1.17(1.16 ,1.19)	3.48E-145
Death	Total PA	0.94(0.93 ,0.96)	2.08E-15
Diabetes	Blue space	0.99(0.97 ,1.01)	1
Diabetes	Current Smoke	1.86(1.77 ,1.96)	3.28E-123
Diabetes	Depression/anxiety	1.51(1.46 ,1.57)	3.65E-111
Diabetes	Diet	0.89(0.87 ,0.90)	1.44E-39
Diabetes	Green space	0.93(0.91 ,0.94)	2.99E-15
Diabetes	IMD scores	1.21(1.19 ,1.23)	7.60E-150
Diabetes	Leisure PA	0.84(0.82 ,0.85)	1.87E-59
Diabetes	NO2	1.10(1.09 ,1.12)	5.96E-31
Diabetes	Noise	1.02(1.00 ,1.03)	1
Diabetes	NOx	1.10(1.08 ,1.11)	8.30E-34
Diabetes	PM10	1.06(1.04 ,1.07)	1.59E-09
Diabetes	PM2.5	1.12(1.11 ,1.14)	3.11E-45
Diabetes	Previous Smoke	1.28(1.24 ,1.33)	2.82E-38
Diabetes	Sedentory behavior	1.15(1.14 ,1.17)	4.74E-82
Diabetes	Sleep duration	0.81(0.78 ,0.84)	3.30E-30
Diabetes	Social isolation	1.10(1.09 ,1.12)	2.95E-32

Supplementary Table 5 Associations between Individual Exposures and Cardiometabolic Diseases using Cox models adjusted with categorized Age and BMI, with significance assessed by two-sided Wald tests and Bonferroni correction.

Outcome	Exposure	HR(95% CI)	Adjusted p-value
Diabetes	Total PA	0.95(0.93 ,0.97)	9.72E-08
Heart Disease	Blue space	1.00(0.99 ,1.01)	1
Heart Disease	Current Smoke	1.56(1.51 ,1.60)	5.10E-180
Heart Disease	Depression/anxiety	1.24(1.21 ,1.26)	4.66E-78
Heart Disease	Diet	0.97(0.96 ,0.98)	1.57E-06
Heart Disease	Green space	0.98(0.97 ,0.99)	2.05E-05
Heart Disease	IMD scores	1.09(1.08 ,1.10)	1.58E-82
Heart Disease	Leisure PA	0.97(0.96 ,0.98)	2.26E-08
Heart Disease	NO2	1.03(1.03 ,1.04)	2.55E-11
Heart Disease	Noise	1.00(0.99 ,1.01)	1
Heart Disease	NOx	1.03(1.02 ,1.04)	1.94E-11
Heart Disease	PM10	1.02(1.01 ,1.03)	0.020670772
Heart Disease	PM2.5	1.04(1.03 ,1.05)	6.01E-14
Heart Disease	Previous Smoke	1.19(1.16 ,1.21)	4.55E-57
Heart Disease	Sedentory behavior	1.06(1.06 ,1.07)	8.59E-39
Heart Disease	Sleep duration	0.90(0.88 ,0.92)	2.34E-24
Heart Disease	Social isolation	1.03(1.02 ,1.04)	6.07E-08
Heart Disease	Total PA	1.00(1.00 ,1.01)	1
Renal Disease	Blue space	1.00(0.99 ,1.01)	1
Renal Disease	Current Smoke	1.64(1.57 ,1.71)	5.35E-126
Renal Disease	Depression/anxiety	1.37(1.33 ,1.41)	2.60E-105
Renal Disease	Diet	0.91(0.90 ,0.93)	1.35E-38
Renal Disease	Green space	0.96(0.95 ,0.98)	2.36E-07
Renal Disease	IMD scores	1.17(1.16 ,1.19)	2.98E-171
Renal Disease	Leisure PA	0.90(0.88 ,0.91)	3.78E-50
Renal Disease	NO2	1.06(1.05 ,1.08)	8.84E-21
Renal Disease	Noise	1.01(0.99 ,1.02)	1
Renal Disease	NOx	1.06(1.05 ,1.08)	1.15E-23
Renal Disease	PM10	1.05(1.04 ,1.06)	7.10E-14
Renal Disease	PM2.5	1.08(1.07 ,1.10)	5.71E-37
Renal Disease	Previous Smoke	1.23(1.19 ,1.26)	1.03E-46
Renal Disease	Sedentory behavior	1.15(1.14 ,1.16)	5.34E-123
Renal Disease	Sleep duration	0.82(0.80 ,0.84)	1.58E-46
Renal Disease	Social isolation	1.09(1.08 ,1.11)	1.91E-45
Renal Disease	Total PA	0.95(0.94 ,0.96)	1.98E-13

Supplementary Table 6 C-index Estimation with Different Parameters in

Outcome	C-Index	nrounds	gamma	max_depth
Death	0.7497	1000	0.7	3
Death	0.7497	1000	0.5	3
Death	0.7496	1000	0.3	3
Death	0.7494	1000	0.7	4
Death	0.7492	1000	0.5	4
Death	0.7492	1000	0.3	4
Death	0.7485	2000	0.5	3
Death	0.7485	2000	0.7	3
Death	0.7484	2000	0.3	3
Death	0.7484	1000	0.7	5
Death	0.7483	1000	0.5	5
Death	0.7480	1000	0.3	5
Death	0.7472	2000	0.5	4
Death	0.7471	2000	0.7	4
Death	0.7470	2000	0.3	4
Death	0.7450	2000	0.7	5
Death	0.7447	2000	0.3	5
Death	0.7445	2000	0.5	5
Heart disease	0.7224	1000	0.3	3
Heart disease	0.7212	2000	0.3	3
Heart disease	0.7224	1000	0.5	3
Heart disease	0.7211	2000	0.5	3
Heart disease	0.7224	1000	0.7	3
Heart disease	0.7213	2000	0.7	3
Heart disease	0.7214	1000	0.3	4
Heart disease	0.7199	2000	0.3	4
Heart disease	0.7214	1000	0.5	4
Heart disease	0.7197	2000	0.5	4
Heart disease	0.7215	1000	0.7	4
Heart disease	0.7198	2000	0.7	4
Heart disease	0.7205	1000	0.3	5
Heart disease	0.7179	2000	0.3	5
Heart disease	0.7202	1000	0.5	5
Heart disease	0.7177	2000	0.5	5
Heart disease	0.7204	1000	0.7	5
Heart disease	0.7178	2000	0.7	5
Cerebrovascular disease	0.7264	1000	0.3	3
Cerebrovascular disease	0.7238	2000	0.3	3
Cerebrovascular disease	0.7264	1000	0.5	3
Cerebrovascular disease	0.7238	2000	0.5	3
Cerebrovascular disease	0.7264	1000	0.7	3
Cerebrovascular disease	0.7239	2000	0.7	3
Cerebrovascular disease	0.7247	1000	0.3	4
Cerebrovascular disease	0.7198	2000	0.3	4
Cerebrovascular disease	0.7244	1000	0.5	4
Cerebrovascular disease	0.7199	2000	0.5	4
Cerebrovascular disease	0.7249	1000	0.7	4
Cerebrovascular disease	0.7229	2000	0.7	4
Cerebrovascular disease	0.7214	1000	0.3	5
Cerebrovascular disease	0.7146	2000	0.3	5
Cerebrovascular disease	0.7215	1000	0.5	5

Supplementary Table 6 C-index Estimation with Different Parameters in

Outcome	C-Index	nrounds	gamma	max_depth
Cerebrovascular disease	0.7140	2000	0.5	5
Cerebrovascular disease	0.7212	1000	0.7	5
Cerebrovascular disease	0.7145	2000	0.7	5
Diabetes	0.7913	1000	0.3	3
Diabetes	0.7895	2000	0.3	3
Diabetes	0.7911	1000	0.5	3
Diabetes	0.7894	2000	0.5	3
Diabetes	0.7912	1000	0.7	3
Diabetes	0.7895	2000	0.7	3
Diabetes	0.7902	1000	0.3	4
Diabetes	0.7865	2000	0.3	4
Diabetes	0.7899	1000	0.5	4
Diabetes	0.7863	2000	0.5	4
Diabetes	0.7899	1000	0.7	4
Diabetes	0.7864	2000	0.7	4
Diabetes	0.7873	1000	0.3	5
Diabetes	0.7824	2000	0.3	5
Diabetes	0.7875	1000	0.5	5
Diabetes	0.7824	2000	0.5	5
Diabetes	0.7871	1000	0.7	5
Diabetes	0.7817	2000	0.7	5
Renal disease	0.7536	1000	0.3	3
Renal disease	0.7523	2000	0.3	3
Renal disease	0.7536	1000	0.5	3
Renal disease	0.7522	2000	0.5	3
Renal disease	0.7535	1000	0.7	3
Renal disease	0.7526	2000	0.7	3
Renal disease	0.7526	1000	0.3	4
Renal disease	0.7502	2000	0.3	4
Renal disease	0.7527	1000	0.5	4
Renal disease	0.7503	2000	0.5	4
Renal disease	0.7527	1000	0.7	4
Renal disease	0.7502	2000	0.7	4
Renal disease	0.7513	1000	0.3	5
Renal disease	0.7473	2000	0.3	5
Renal disease	0.7511	1000	0.5	5
Renal disease	0.7472	2000	0.5	5
Renal disease	0.7512	1000	0.7	5
Renal disease	0.7476	2000	0.7	5

Supplementary Table 7 Association Between Co-Exposure Patterns and Cardiometabolic Diseases in the Healthy Subgroup, with significance assessed by two-sided Wald tests.

Exposure pattern	Disease	HR (95%CI)	p-value
Air and noise pollution	Death	1.18 (1.11 ,1.26)	3.23E-07
Air and noise pollution	Heart Disease	1.06 (1.01 ,1.11)	0.010916848
Air and noise pollution	Cerebrovascular Disease	1.21 (1.12 ,1.31)	3.60E-06
Air and noise pollution	Diabetes	1.19 (1.10 ,1.28)	1.27E-05
Air and noise pollution	Renal Disease	1.10 (1.03 ,1.18)	0.00284942
Blue and green spaces	Death	0.95 (0.91 ,0.99)	0.008249624
Blue and green spaces	Heart Disease	0.97 (0.94 ,0.99)	0.011509133
Blue and green spaces	Cerebrovascular Disease	0.98 (0.93 ,1.03)	0.383966921
Blue and green spaces	Diabetes	0.89 (0.84 ,0.93)	5.27E-06
Blue and green spaces	Renal Disease	0.98 (0.94 ,1.02)	0.299467609
Health behaviors	Death	0.91 (0.86 ,0.96)	0.000568729
Health behaviors	Heart Disease	1.04 (1.00 ,1.07)	0.045638832
Health behaviors	Cerebrovascular Disease	1.05 (0.98 ,1.12)	0.140967908
Health behaviors	Diabetes	0.88 (0.81 ,0.94)	0.00049517
Health behaviors	Renal Disease	0.93 (0.88 ,0.99)	0.017319313
Social deprivation	Death	1.32 (1.27 ,1.37)	1.04E-42
Social deprivation	Heart Disease	1.14 (1.11 ,1.18)	1.65E-21
Social deprivation	Cerebrovascular Disease	1.34 (1.27 ,1.40)	2.31E-30
Social deprivation	Diabetes	1.32 (1.26 ,1.39)	2.39E-32
Social deprivation	Renal Disease	1.28 (1.23 ,1.33)	6.18E-34

Supplementary Table 8 Association Between Proteomic Signature and Cardiometabolic Diseases in the Healthy Subgroup, with significance assessed by two-sided Wald tests

Exposure pattern	Disease	HR (95%CI)	p-value
Social deprivation	Death	1.59 (1.53 ,1.65)	1.42E-130
Social deprivation	Heart Disease	1.27 (1.23 ,1.31)	2.52E-56
Social deprivation	Cerebrovascular Disease	1.30 (1.23 ,1.36)	8.17E-23
Social deprivation	Diabetes	1.49 (1.42 ,1.57)	7.66E-52
Social deprivation	Renal Disease	1.49 (1.43 ,1.55)	2.54E-88
Blue and green spaces	Death	0.86 (0.82 ,0.90)	3.34E-11
Blue and green spaces	Heart Disease	0.97 (0.93 ,1.00)	0.034200426
Blue and green spaces	Cerebrovascular Disease	0.95 (0.90 ,1.01)	0.105791052
Blue and green spaces	Diabetes	0.82 (0.77 ,0.87)	3.62E-11
Blue and green spaces	Renal Disease	1.07 (1.03 ,1.12)	0.002094257
Health behaviors	Death	0.97 (0.92 ,1.02)	0.284274542
Health behaviors	Heart Disease	1.04 (1.01 ,1.08)	0.025499251
Health behaviors	Cerebrovascular Disease	0.96 (0.90 ,1.03)	0.237939581
Health behaviors	Diabetes	0.83 (0.77 ,0.88)	2.30E-08
Health behaviors	Renal Disease	0.94 (0.89 ,0.99)	0.017443576
Air and noise pollution	Death	1.64 (1.57 ,1.71)	5.82E-115
Air and noise pollution	Heart Disease	1.3 (1.25 ,1.34)	1.28E-54
Air and noise pollution	Cerebrovascular Disease	1.32 (1.25 ,1.40)	6.60E-22
Air and noise pollution	Diabetes	1.45 (1.37 ,1.54)	3.26E-36
Air and noise pollution	Renal Disease	1.62 (1.55 ,1.70)	2.16E-107

Supplementary Table 9 Association Between the Proteomic Signature and Cardiometabolic Diseases with Different Exclusion Windows, with significance assessed by two-sided Wald tests

Exposure pattern signature	Outcome	HR,95%CI	p-value	Exclusion window
Social deprivation	Death	1.685(1.634,1.737)	1.0567E-246	60 days
Social deprivation	Heart Disease	1.289(1.254,1.326)	7.6685E-72	60 days
Social deprivation	Cerebrovascular Disease	1.347(1.289,1.407)	8.5506E-41	60 days
Social deprivation	Diabetes	1.475(1.408,1.545)	2.1465E-60	60 days
Social deprivation	Renal Disease	1.539(1.49,1.59)	1.8296E-149	60 days
Blue and green spaces	Death	0.854(0.823,0.886)	5.0080E-17	60 days
Blue and green spaces	Heart Disease	0.97(0.941,0.999)	4.4708E-02	60 days
Blue and green spaces	Cerebrovascular Disease	0.954(0.909,1.002)	6.1951E-02	60 days
Blue and green spaces	Diabetes	0.817(0.776,0.86)	1.7676E-14	60 days
Blue and green spaces	Renal Disease	1.046(1.008,1.087)	1.8789E-02	60 days
Health behaviors	Death	0.908(0.87,0.948)	1.0237E-05	60 days
Health behaviors	Heart Disease	1.019(0.985,1.055)	2.7682E-01	60 days
Health behaviors	Cerebrovascular Disease	0.959(0.906,1.015)	1.4701E-01	60 days
Health behaviors	Diabetes	0.802(0.755,0.851)	4.0883E-13	60 days
Health behaviors	Renal Disease	0.901(0.862,0.942)	3.5839E-06	60 days
Air and noise pollution	Death	1.765(1.706,1.826)	5.2604E-236	60 days
Air and noise pollution	Heart Disease	1.321(1.282,1.362)	8.9500E-73	60 days
Air and noise pollution	Cerebrovascular Disease	1.382(1.317,1.449)	1.6911E-40	60 days
Air and noise pollution	Diabetes	1.434(1.362,1.51)	1.9271E-42	60 days
Air and noise pollution	Renal Disease	1.679(1.62,1.739)	2.4322E-179	60 days
Social deprivation	Death	1.665(1.614,1.718)	2.0465E-226	1 year
Social deprivation	Heart Disease	1.285(1.249,1.322)	7.2234E-67	1 year
Social deprivation	Cerebrovascular Disease	1.35(1.292,1.411)	1.7262E-40	1 year
Social deprivation	Diabetes	1.473(1.405,1.543)	5.3631E-59	1 year
Social deprivation	Renal Disease	1.533(1.483,1.585)	2.5568E-140	1 year
Blue and green spaces	Death	0.862(0.83,0.895)	6.9539E-15	1 year
Blue and green spaces	Heart Disease	0.97(0.941,1.001)	5.5795E-02	1 year
Blue and green spaces	Cerebrovascular Disease	0.958(0.912,1.007)	9.2296E-02	1 year
Blue and green spaces	Diabetes	0.82(0.778,0.864)	7.3093E-14	1 year
Blue and green spaces	Renal Disease	1.045(1.005,1.086)	2.6753E-02	1 year
Health behaviors	Death	0.916(0.877,0.957)	7.1247E-05	1 year
Health behaviors	Heart Disease	1.025(0.99,1.062)	1.6577E-01	1 year
Health behaviors	Cerebrovascular Disease	0.954(0.901,1.01)	1.0582E-01	1 year
Health behaviors	Diabetes	0.802(0.755,0.852)	6.8686E-13	1 year
Health behaviors	Renal Disease	0.906(0.866,0.948)	1.6941E-05	1 year
Air and noise pollution	Death	1.743(1.684,1.804)	4.7169E-217	1 year
Air and noise pollution	Heart Disease	1.317(1.276,1.358)	5.7331E-68	1 year
Air and noise pollution	Cerebrovascular Disease	1.386(1.32,1.454)	4.2104E-40	1 year
Air and noise pollution	Diabetes	1.435(1.362,1.512)	5.9132E-42	1 year
Air and noise pollution	Renal Disease	1.67(1.611,1.732)	2.3521E-168	1 year

Supplementary Table 10 Association Between the Proteomic Signature and Cardiometabolic Diseases adjusted for renal function, with significance assessed by two-sided Wald tests

Proteomic signature	Disease	HR(95%CI)	P value	Adjustment of co-exposure pattern
Social deprivation	Death	1.68(1.62,1.73)	6.50E-200	Adjusted
Social deprivation	Heart Disease	1.29(1.25,1.33)	9.25E-62	Adjusted
Social deprivation	Cerebrovascular Disease	1.34(1.27,1.4)	1.22E-32	Adjusted
Social deprivation	Diabetes	1.47(1.4,1.54)	3.64E-50	Adjusted
Social deprivation	Renal Disease	1.49(1.44,1.55)	8.05E-106	Adjusted
Blue and green spaces	Death	0.85(0.82,0.88)	8.82E-16	Adjusted
Blue and green spaces	Heart Disease	0.97(0.94,1)	0.06880956	Adjusted
Blue and green spaces	Cerebrovascular Disease	0.94(0.89,0.99)	0.0228572	Adjusted
Blue and green spaces	Diabetes	0.83(0.79,0.88)	1.21E-10	Adjusted
Blue and green spaces	Renal Disease	1.04(1,1.08)	0.07164515	Adjusted
Health behaviors	Death	0.92(0.88,0.96)	0.00037618	Adjusted
Health behaviors	Heart Disease	1.02(0.99,1.06)	0.20951375	Adjusted
Health behaviors	Cerebrovascular Disease	0.95(0.89,1.01)	0.11453235	Adjusted
Health behaviors	Diabetes	0.8(0.75,0.86)	4.51E-11	Adjusted
Health behaviors	Renal Disease	0.95(0.9,0.99)	0.02373489	Adjusted
Air and noise pollution	Death	1.74(1.68,1.81)	8.97E-185	Adjusted
Air and noise pollution	Heart Disease	1.32(1.28,1.37)	4.66E-62	Adjusted
Air and noise pollution	Cerebrovascular Disease	1.36(1.29,1.43)	1.06E-30	Adjusted
Air and noise pollution	Diabetes	1.44(1.36,1.52)	1.33E-36	Adjusted
Air and noise pollution	Renal Disease	1.6(1.54,1.67)	1.37E-124	Adjusted
Social deprivation	Death	1.68(1.63,1.74)	1.34E-213	Unadjusted
Social deprivation	Heart Disease	1.29(1.25,1.33)	2.27E-62	Unadjusted
Social deprivation	Cerebrovascular Disease	1.35(1.28,1.41)	2.22E-35	Unadjusted
Social deprivation	Diabetes	1.48(1.41,1.56)	4.70E-55	Unadjusted
Social deprivation	Renal Disease	1.5(1.45,1.55)	1.82E-112	Unadjusted
Blue and green spaces	Death	0.84(0.81,0.88)	3.01E-17	Unadjusted
Blue and green spaces	Heart Disease	0.97(0.94,1)	0.06138629	Unadjusted
Blue and green spaces	Cerebrovascular Disease	0.94(0.89,0.99)	0.02392761	Unadjusted
Blue and green spaces	Diabetes	0.83(0.78,0.88)	2.20E-11	Unadjusted
Blue and green spaces	Renal Disease	1.03(0.99,1.08)	0.1239709	Unadjusted
Health behaviors	Death	0.91(0.87,0.95)	7.33E-05	Unadjusted
Health behaviors	Heart Disease	1.03(0.99,1.07)	0.14990288	Unadjusted
Health behaviors	Cerebrovascular Disease	0.96(0.9,1.01)	0.13727451	Unadjusted
Health behaviors	Diabetes	0.8(0.75,0.86)	2.20E-11	Unadjusted
Health behaviors	Renal Disease	0.93(0.89,0.98)	0.00456117	Unadjusted
Air and noise pollution	Death	1.75(1.69,1.81)	7.41E-193	Unadjusted
Air and noise pollution	Heart Disease	1.32(1.28,1.36)	9.01E-63	Unadjusted
Air and noise pollution	Cerebrovascular Disease	1.37(1.3,1.44)	7.72E-33	Unadjusted
Air and noise pollution	Diabetes	1.45(1.37,1.53)	3.57E-39	Unadjusted
Air and noise pollution	Renal Disease	1.61(1.55,1.67)	1.32E-128	Unadjusted

Supplementary Table 11 Prevalence of Pre-existing diseases in exposure pattern analysis, n(%)

Analysis data set	Heart disease	Cerebrovascular disease	Renal Diease	Diabetes
Heart	0(0)	4206(1.25)	3790(1.13)	13830(4.11)
Cerebrovascular	20691(5.86)	0(0)	4679(1.32)	16593(4.7)
Renal	20903(5.91)	5307(1.5)	0(0)	16419(4.64)
Diabetes	18580(5.44)	4858(1.42)	4056(1.19)	0(0)
Death	22093(6.16)	5608(1.56)	4980(1.39)	17343(4.83)

Supplementary Table 12 Prevalence of Pre-existing diseases in proteomic analysis, n(%)

Analysis data set	Heart disease	Cerebrovascular disease	Renal Diease	Diabetes
Heart	0(0)	470(1.34)	509(1.45)	1494(4.26)
Cerebrovascular	2439(6.59)	0(0)	670(1.81)	1832(4.95)
Renal	2416(6.54)	608(1.64)	0(0)	1788(4.84)
Diabetes	2192(6.13)	561(1.57)	579(1.62)	0(0)
Death	2631(6.98)	662(1.76)	724(1.92)	1933(5.13)

Supplementary Reference

1. UK Biobank: Protocol for a large-scale prospective epidemiological resource (Protocol No: UKBB-PROT-09-06). UK Biobank Coordinating Centre. 2006.
2. Vienneau D, de Hoogh K, Bechle MJ, Beelen R, van Donkelaar A, Martin RV, Millet DB, Hoek G, Marshall JD. Western European land use regression incorporating satellite- and ground-based measurements of NO2 and PM10. Environ Sci Technol. 2013;47(23):13555-64.
