# Supplementary material for: Personal, social, and natural co-exposure pattern and plasma proteins in cardiometabolic diseases
Source: Nat Commun. 2025 Nov 25;16:10498. doi: 10.1038/s41467-025-65516-2 (PMC12647215; doi:10.1038/s41467-025-65516-2)
Supplement: Supplementary file 2 — Description of Additional Supplementary Information [file 41467_2025_65516_MOESM2_ESM.pdf]

Description of Additional Supplementary Files

File Name: Supplementary Data 1
Description: Full protein names and description.

File Name: Supplementary Data 2
Description: Association Between Proteins and Cardiometabolic Diseases in Exposure Pattern Subgroups, assessed using twosided Wald tests with Bonferroni correction for multiple comparisons.

File Name: Supplementary Data 3
Description: Proteins' signature weights and mean concentration in specific exposure pattern group.

File Name: Supplementary Data 4
Description: Mediation Effects of Feature Proteins on Cardiometabolic Diseases, assessed using two-sided Z-tests based on the delta method and adjusted for multiple comparisons using the Bonferroni method.

File Name: Supplementary Data 5
Description: Full Mediation Analysis of Feature Proteins adjusted for renal function, assessed using two-sided Z-tests based on the delta method and adjusted for multiple comparisons using the Bonferroni method.
