# Supplementary material for: Personal, social, and natural co-exposure pattern and plasma proteins in cardiometabolic diseases
Source: Nat Commun. 2025 Nov 25;16:10498. doi: 10.1038/s41467-025-65516-2 (PMC12647215; doi:10.1038/s41467-025-65516-2)
Supplement: Supplementary file 8 — Reporting Summary [file 41467_2025_65516_MOESM8_ESM.pdf]

nature portfolio

Corresponding author(s): JZ, XX, BG, XZ

Last updated by author(s): Sep 7, 2025

Reporting Summary

Nature Portfolio wishes to improve the reproducibility of the work that we publish. This form provides structure for consistency and transparency in reporting. For further information on Nature Portfolio policies, see our Editorial Policies and the Editorial Policy Checklist.

Statistics

For all statistical analyses, confirm that the following items are present in the figure legend, table legend, main text, or Methods section.

n/a	Confirmed	
☐	☒	The exact sample size (*n*) for each experimental group/condition, given as a discrete number and unit of measurement
☐	☒	A statement on whether measurements were taken from distinct samples or whether the same sample was measured repeatedly
☐	☒	The statistical test(s) used AND whether they are one- or two-sided<br>*Only common tests should be described solely by name; describe more complex techniques in the Methods section.*
☐	☒	A description of all covariates tested
☐	☒	A description of any assumptions or corrections, such as tests of normality and adjustment for multiple comparisons
☐	☒	A full description of the statistical parameters including central tendency (e.g. means) or other basic estimates (e.g. regression coefficient) AND variation (e.g. standard deviation) or associated estimates of uncertainty (e.g. confidence intervals)
☐	☒	For null hypothesis testing, the test statistic (e.g. *F*, *t*, *r*) with confidence intervals, effect sizes, degrees of freedom and *P* value noted<br>*Give P values as exact values whenever suitable.*
☒	☐	For Bayesian analysis, information on the choice of priors and Markov chain Monte Carlo settings
☒	☐	For hierarchical and complex designs, identification of the appropriate level for tests and full reporting of outcomes
☐	☒	Estimates of effect sizes (e.g. Cohen's *d*, Pearson's *r*), indicating how they were calculated

Our web collection on statistics for biologists contains articles on many of the points above.

Software and code

Policy information about availability of computer code

Data collection	No software used
Data analysis	Relevant analyses in this study were conducted using R version 4.4.1. Codes for the analyses are available at https://github.com/Vi-1105/ExposurePatternCMD/tree/main.

For manuscripts utilizing custom algorithms or software that are central to the research but not yet described in published literature, software must be made available to editors and reviewers. We strongly encourage code deposition in a community repository (e.g. GitHub). See the Nature Portfolio guidelines for submitting code & software for further information.

Data

Policy information about availability of data

All manuscripts must include a data availability statement. This statement should provide the following information, where applicable:

- Accession codes, unique identifiers, or web links for publicly available datasets
- A description of any restrictions on data availability
- For clinical datasets or third party data, please ensure that the statement adheres to our policy

This study is based on data from the UK Biobank (Application Number 117185). Detailed information about data availability in UK Biobank can be accessed online at www.ukbiobank.co.uk. Source data are provided with this paper.

Research involving human participants, their data, or biological material

Policy information about studies with human participants or human data. See also policy information about sex, gender (identity/presentation), and sexual orientation and race, ethnicity and racism.

Reporting on sex and gender	Sex was categorized as male and female based on self-reporting, with consent obtained for sharing individual-level data.
Reporting on race, ethnicity, or other socially relevant groupings	Ethnicity was classified as White, Mixed, Asian or Asian British, Black or Black British, Chinese, and Other, based on self-reporting; we ensured that ethnicity was not used as a proxy for socioeconomic status and provided clear definitions for each category while controlling for confounding variables to ensure accurate analyses.
Population characteristics	Participants ranged in age from 37 to 73 years and included diverse genotype information, with clinical diagnoses indicating that some participants had conditions such as diabetes and hypertension.
Recruitment	UKB recruitment was centrally coordinated through population-based registers, such as those maintained by the NHS, to identify and invite eligible individuals within reasonable proximity to an assessment center.
Ethics oversight	The North West Multi-Centre Research Ethics Committee as a Research Tissue Bank approved the UKB.

Note that full information on the approval of the study protocol must also be provided in the manuscript.

Field-specific reporting

Please select the one below that is the best fit for your research. If you are not sure, read the appropriate sections before making your selection.

☒ Life sciences ☐ Behavioural & social sciences ☐ Ecological, evolutionary & environmental sciences

For a reference copy of the document with all sections, see nature.com/documents/nr-reporting-summary-flat.pdf

Life sciences study design

All studies must disclose on these points even when the disclosure is negative.

Sample size	This study was based on the UK Biobank cohort, which includes 502,394 baseline participants. After applying exclusion criteria, 366,261 participants were retained for exposure pattern clustering analysis. For cardiometabolic disease association analyses, 358,872 participants with complete follow-up data were included, forming disease-specific sub-cohorts: heart disease (n = 336,778; 43,173 incident cases), cerebrovascular disease (n = 353,264; 14,863 cases), renal disease (n = 353,892; 24,720 cases), diabetes (n = 341,529; 13,940 cases), and all-cause mortality (n = 358,872; 23,902 deaths).<br>A total of 37,687 participants with available proteomic data were included in subsequent protein association analyses. Disease-specific sample sizes in this sub-cohort were: heart disease (n = 35,056), cerebrovascular disease (n = 37,025), renal disease (n = 36,963), diabetes (n = 35,754), and mortality (n = 37,687).<br>Sample sizes were based on data availability, and the large cohort size supports the validity and robustness of the analytical results.
Data exclusions	Data exclusions were applied based on pre-specified criteria to ensure analytical validity. Participants with missing exposure variables (n = 136,133) were excluded to guarantee completeness for exposure pattern clustering analysis. An additional 7,389 individuals were excluded due to missing key covariates or ethnicity data, which are essential for statistical adjustment in cardiometabolic disease analyses. To minimize reverse causation, participants with the target diseases or who had died at baseline were excluded from corresponding outcome analyses, including 22,094 for heart disease, 5,608 for cerebrovascular disease, 4,980 for renal disease, and 17,343 for diabetes. Furthermore, 321,185 participants were excluded from the proteomic analyses due to missing protein data. These exclusions ensured data quality, reduced bias, and allowed for the accurate definition of disease-free populations at baseline.
Replication	The associations between exposure patterns and cardiometabolic diseases (CMDs) were replicated in over 300,000 participants with complete exposure data. Associations between protein signatures and CMDs were further validated in a proteomic sub-cohort of over 30,000 participants. Additionally, sensitivity analyses excluding individuals with baseline CMDs and those with early-onset events (within 60 days or 1 year after baseline) consistently supported the robustness of the observed associations. All replication attempts were successful.
Randomization	We don't have group allocation.
Blinding	We don't have group allocation.

Reporting for specific materials, systems and methods

We require information from authors about some types of materials, experimental systems and methods used in many studies. Here, indicate whether each material, system or method listed is relevant to your study. If you are not sure if a list item applies to your research, read the appropriate section before selecting a response.

Materials & experimental systems

n/a	Involved in the study
☒	☐ Antibodies
☒	☐ Eukaryotic cell lines
☒	☐ Palaeontology and archaeology
☒	☐ Animals and other organisms
☒	☐ Clinical data
☒	☐ Dual use research of concern
☒	☐ Plants

Methods

n/a	Involved in the study
☒	☐ ChIP-seq
☒	☐ Flow cytometry
☒	☐ MRI-based neuroimaging

Plants

Seed stocks	This study does not involve.
Novel plant genotypes	This study does not involve.
Authentication	This study does not involve.
